# Reviewing Interspecies Interactions as a Driving Force Affecting the Community Structure in Lakes via Cyanotoxins

**DOI:** 10.3390/microorganisms9081583

**Published:** 2021-07-25

**Authors:** Azam Omidi, Stephan Pflugmacher, Aaron Kaplan, Young Jun Kim, Maranda Esterhuizen

**Affiliations:** 1 Chair Ecological Impact Research and Ecotoxicology, Technische Universität Berlin, 10587 Berlin, Germany; azam.omidi@gmail.com; 2 Clayton H. Riddell Faculty of Environment, Earth, and Resources, University of Manitoba, Wallace Bldg., 125 Dysart Rd, Winnipeg, MB R3T 2N2, Canada; Stephan.PflugmacherLima@umanitoba.ca; 3 Department of Plant and Environmental Sciences, Edmond J. Safra Campus, The Hebrew University of Jerusalem, Jerusalem 9190401, Israel; aaron.kaplan@mail.huji.ac.il; 4 Joint Laboratory of Applied Ecotoxicology, Korean Institute of Science and Technology Europe (KIST), Campus 7.1, 66123 Saarbrücken, Germany; youngjunkim@kist-europe.de; 5 Ecosystems and Environment Research Programme, Faculty of Biological and Environmental Sciences, University of Helsinki, Niemenkatu 73, 15140 Lahti, Finland; 6 Finland and Helsinki Institute of Sustainability Science (HELSUS), Fabianinkatu 33, 00014 Helsinki, Finland

**Keywords:** cyanobacteria, cyanotoxins, interspecies interactions, allelopathy, lake ecosystems

## Abstract

The escalating occurrence of toxic cyanobacterial blooms worldwide is a matter of concern. Global warming and eutrophication play a major role in the regularity of cyanobacterial blooms, which has noticeably shifted towards the predomination of toxic populations. Therefore, understanding the effects of cyanobacterial toxins in aquatic ecosystems and their advantages to the producers are of growing interest. In this paper, the current literature is critically reviewed to provide further insights into the ecological contribution of cyanotoxins in the variation of the lake community diversity and structure through interspecies interplay. The most commonly detected and studied cyanobacterial toxins, namely the microcystins, anatoxins, saxitoxins, cylindrospermopsins and β-N-methylamino-L-alanine, and their ecotoxicity on various trophic levels are discussed. This work addresses the environmental characterization of pure toxins, toxin-containing crude extracts and filtrates of single and mixed cultures in interspecies interactions by inducing different physiological and metabolic responses. More data on these interactions under natural conditions and laboratory-based studies using direct co-cultivation approaches will provide more substantial information on the consequences of cyanotoxins in the natural ecosystem. This review is beneficial for understanding cyanotoxin-mediated interspecies interactions, developing bloom mitigation technologies and robustly assessing the hazards posed by toxin-producing cyanobacteria to humans and other organisms.

## 1. Introduction

In aquatic ecosystems, the primary producers, such as cyanobacteria, algae, bacteria and plants, often function as foundation as well as keystone species [1] because they drive primary production, play a pivotal role in food webs and nutrient cycling and create habitats for other species. Several factors can influence the structure and biodiversity over time within a community, such as abiotic and biotic parameters, frequency, level of disturbances, chance events and the interactions between species, including via allelopathy [2,3,4,5,6].

**Figure 1:**
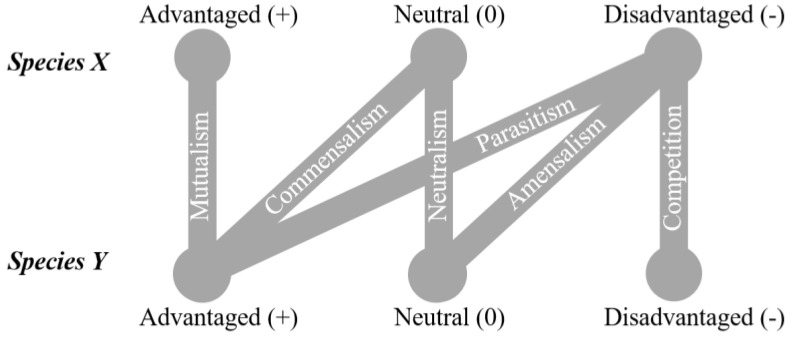
Long-terms interspecies interactions. Lines indicate how each species is influenced by the other.

Species in a community may interact with each other in various ways, forming a biocoenosis. The interactions can be interspecies or intraspecies, having a strong influence on the evolution of the species, and often the partners coevolve [7]. Traditionally, these interactions have been classified and termed as follows (Figure 1): Neutralism (0/0), mutualism (+/+), Competition (-/-), Commensalism (+/0), Amensalism (-/0), parasitism (+/-) and Predation (+/-) [8,9]. These interactions, both as inter- and intraspecies exchanges, change with both time and location and are dynamic based on the prevailing conditions being either beneficial, inhibitory or commensal [10,11]. 

Cyanobacteria frequently form blooms and dominate the aquatic ecosystem community. These blooms influence water quality through the sheer quantity of cyanobacterial cells and the release of secondary metabolites, including toxic compounds, thereby affecting the rest of the biological community and their ecosystem functions and services [12,13]. The frequency and prevalence of blooms are increasing, likely due to anthropogenic effects [14], leading to excess nutrients and global warming [15]. Interestingly, there are indeed coexisting antagonists in these environments where blooms occur, such as cyanolytic bacteria and grazers [16,17,18]. Regardless, cyanobacterial blooms annually arise globally [19], often in succession with green algae and diatoms [20,21,22]. It is essential to recognize that conditions associated with blooms may lead to reduced competition or predation on the taxa forming the blooms [15]. However, the exact mechanism used to achieve dominance and the interactions involved are poorly understood. Allelopathic interactions could cause cyanobacterial dominance as allelochemicals could allow cyanobacteria to outcompete other phytoplankton. As the frequency of cyanobacterial blooms is increasing worldwide, their allelopathic effects on other aquatic organisms are attracting growing attention. Considering the major societal and economic impacts of cyanobacterial blooms, information regarding allelopathic interspecies interactions could also be vital in developing methods to control blooms development.

Over the past decades, more than 2000 cyanobacterial secondary metabolites have been reported [23]. Previous studies suggested the involvement of the other secondary metabolites rather than or along with cyanotoxins in the allelopathic effects [24,25]. Two well-characterized allelochemicals, fischerellin produced by *Fischerella muscicola* [26] and cyanobacterin produced by *Scytonema hofmanni* [27], have been implicated in photosynthesis inhibition of co-occurring species. In addition, *Microcystis aeruginosa* was reported to release linoleic acid, which inhibits the growth of *Chlorella vulgaris* and photosynthesis, carbon metabolism and amino acid metabolism in *Auxenochlorella pyrenoidosa* (previously *Chlorella pyrenoidosa*) [28,29]. 

Studies on interspecies interactions involving cyanobacteria mostly focused on previously recognized toxins [30,31,32]. Cyanotoxins are very diverse bioactive secondary metabolites, which are released into aquatic environments at high concentrations when cyanobacterial blooms occur and subsequently collapse (as reviewed by Wiegand and Pflugmacher [33]). Previous publications have shown that some cyanotoxins have allelopathic properties as they influence other members of the aquatic community (reviewed by Leão et al. [34]). Understanding the effects of cyanotoxins on other cyanobacteria could further elucidate the role of toxins in intra- and interspecies interactions, as well as the possible advantages of toxin production [35]. Keating [36,37] proposed that allelopathic interaction through toxins could aid in cyanobacterial dominance, i.e., bloom formation, triggering succession. 

*Dolichospermum circinale* (previously *Anabaena circinalis*), *Aphanizomenon flos-aquae*, *Raphidiopsis raciborskii* (previously *Cylindrospermopsis raciborskii*) and *M. aeruginosa* have been shown to impact other phytoplankton via their toxins [38,39]; e.g., by suppressing grazing, growth and reproduction of antagonists leading to mortality in some cases [40,41,42,43,44,45]. However, it is unknown if all so-called “toxins” play a role as allelopathic biomolecules benefitting the producer. Considering the high energy cost of toxins production, the producer should gain an enormous payoff. 

This review sets out to collect and assimilate the current knowledge regarding the allelopathic interactions of the major cyanotoxin groups and how they could serve as an advantage for cyanobacteria in aquatic communities by assessing the adverse effects reported on biota, which co-occur with cyanobacteria in aquatic environments.

## 2. Cyanotoxins and Interspecies Interplay

### 2.1. Microcystins (MCs)

The microcystins (MCs) are the most abundant and studied cyanotoxins. To date, 286 variants of MCs have been identified [46]. However, beyond MCs a wide variety of other toxic metabolites such as cyanopeptolins, anabaenopeptins, aerucyclamides, aeruginosins and microginins have been reported which pose an ecotoxicological risks and need to receive more attention in future studies [47,48]. 

MCs are produced by the globally distributed genera *Microcystis*, *Anabaena*, *Aphanizomenon*, *Planktothrix* and *Oscillatoria*. *Microcystis* is the most frequently reported bloom-forming cyanobacteria in freshwater ecosystems [19,46,47,48]. Previous studies suggested that in addition to their toxicity, MCs play a major role as allelopathic compounds. Its presence in the media consequent on the lysis of cells “reports” to the rest of the cells that the population is under stress, upregulating microcystin biosynthesis [49]. By binding to specific proteins within the cells, it protects *Microcystis* against oxidative stress [50] and alters cyanobacterial metabolism [51].

#### 2.1.1. MCs and Heterotrophic Bacteria 

Based on geological evidence, Tomescu et al. [52] suggested that interspecies interactions between cyanobacteria and heterotrophs date back 440 million years. In aquatic habitats, cyanobacteria are commonly associated with heterotrophic bacteria [53,54,55], where they communicate and interact with each other within their microenvironments in different ways [56]. Studies by Weiss and co-authors indicated that *M. aeruginosa* and *Aeromonas veronii* recognized and responded to the presence of each other and that secondary metabolites such as lumichrome and others [55,57] are involved. In the aquatic ecosystem, cyanobacteria provide carbon and oxygen and facilitate nutrient exchange such as nitrogen and phosphorus cycling in exchange for key metabolites from the heterotrophic consorts in a mutualistic relationship [58,59,60]. However, the microbial assemblage has been shown to be both positively and negatively influenced by the presence of cyanobacteria. Recent studies showed that the microbial community associated with cyanobacterial blooms is significantly different under non-bloom conditions [61,62]. Moreover, the bacterial composition at pre-bloom, bloom and post-bloom stages was reported to be organized into clearly distinctive cyanobacterial-related microbial groups [63,64]. *Microcystis* blooms, in particular, were found to affect the eukaryotic plankton abundance strongly [28,62]. The decomposition of *Microcystis* blooms increased the amount of dissolved organic matter (DOM), such as carbon and nitrogen sources, within its phycosphere, which contributes to shifts in the bacterial community structure [65,66,67]. Additionally, it should be considered that MCs are contained intracellularly and thus whether MCs as an allelochemical would benefit the producing cyanobacterium when released after bloom collapse. Upon toxic bloom senescence, toxins are released to the surrounding waters, and the presence of extracellular MCs may further drive changes in the bacterial assemblage through allelopathic interactions. For example, the abundance of MC-degrading bacteria (reviewed by Li et al. [68]) increased in accordance with enhanced MC release [69,70,71], resulting in a decreased concentration of dissolved MCs. The presence of MC-degrading bacteria has been reported all year round; however, they positively proliferated in correlation to MC release during and after decaying of summer/autumn toxic cyanobacterial blooms [69,70]. Understanding the factors affecting the composition and concentration of MC degrading bacteria could aid in the development of biotechnological techniques towards toxin remediation from contaminated sites.

The capability of MCs to penetrate the cell membranes of both gram-negative and gram-positive bacteria was reported by Yang et al. [72], where exposure of *Escherichia coli* and *Bacillus subtilis* (isolated and purified from cyanobacterial blooms in Lake Dianchi, China) to MC-RR promoted the bacteriolytic activity of lysozyme through the increased permeability of the cellular outer membrane. The study by Miguéns and Valério [73] further indicated that different variants of MCs (MC-LR, MC-RR and MC-YR) at various concentrations (1, 10 and 1000 nM) significantly reduced the growth of heterotrophic bacteria (phyla: Firmicutes, Actinobacteria, Bacteroidetes, β-Proteobacteria and γ-Proteobacteria), which were isolated from both cyanobacteria contaminated and non-contaminated Portuguese freshwater reservoirs. However, as no differences in the growth reduction of either MC-treated bacteria (co-occurred with blooms and bloom-free species) were observed, the possibility of bacterial adaptation to MCs with pre-exposure was rejected.

Field studies in Lake Taihu, China, demonstrated that blooms negatively affected bacterial diversity and richness [74,75]. It was reported that the diversity of the bacterial community composition, dominated by Actinobacteria, β- and α-Proteobacteria, was reduced in the presence of blooms of MC-producing *Microcystis* sp. in summer and autumn seasons [76]. The biodiversity was negatively related to *Microcystis* abundance and the intracellular MC concentration since the lowest diversity was observed in October when the highest concentration of MC was detected. However, the lower concentration of extracellular MC-LR in October suggested resource competition and environmental changes caused by the proliferation of cyanobacteria rather than altering the microbial community structure in the lake by MC. These data are of immense importance to our understanding of the bacterial-*Microcystis* interaction, a topic that should be further explored. However, information regarding the effects of the declining bacterial abundance on the intensity of the *Microcystis* bloom, toxin production and fate is lacking. 

The relationship between cyanobacteria and heterotrophic bacteria has been described as mutualistic, especially pertaining to nitrogen cycling [11]. A balanced relationship between cyanobacteria and heterotrophic bacteria is necessary to maintain ecological stability [77]. An imbalance of this relationship can bring about cyanobacterial blooms besides the already proved influence of environmental conditions on this relationship [78,79]. It is important to investigate what additional effects cyanobacterial secondary metabolites have on the heterotrophic bacteria to develop cyanobacteria bloom control techniques. 

#### 2.1.2. MCs and Phytoplankton, Including Other Cyanobacteria

In nature, MC-LR has been reported to influence the biodiversity of phytoplankton communities negatively; Tai Lake, China, is just one example [80]. Using 18S ribosomal DNA sequencing, Xue et al. [81] reported that the eukaryotic plankton community composition was significantly altered following a cyanobacterial bloom event, dominated by *M. aeruginosa*, mostly affecting the keystone taxa, whereas the diversity of abundant plankton was hardly affected. The data suggest that cyanobacterial blooms influence the overall aquatic ecosystem functioning by affecting the community turnover of other phytoplankton and further zooplankton communities [82,83]. Advances in molecular techniques and meta-omics data could help further understand the relationships between aquatic species in their natural environment, considering the complex myriad of factors involved and facilitate mining and identification of interspecies molecular events [84].

Singh et al. [85] demonstrated that the purified MC-LR (25 µg mL^−1^) inhibited the growth of *Nostoc muscorum* and *Anabaena* BT1 associated with a significant decrease in CO_2_ uptake, O_2_ evolution and nitrogen fixation activities. MC-containing filtrates from *M. aeruginosa* LE 3 reduced anatoxin (ATX) production and N_2_-fixation in *Trichormus* *variabilis* UTEX B 377 (previously *Anabaena variabilis* UTEX B 377) as well; thus, *Microcystis* gained a comparative advantage [32]. In the co-cultivations of *Anabaena* (*Nostoc*) sp. PCC 7120 with toxic *M. aeruginosa* (PCC 7806) and non-toxic *Microcystis wesenbergii* (FACHB-929), respectively, the toxic strain was the more dominant competitor [86]. An antagonistic relationship between *Microcystis* and cyanobacterium *Synechococcus* was also observed, where *Synechococcus* dominated the blooms in western Lake Erie, U.S., when the abundance of toxic *Microcystis* was lower [87], possibly because MC-RR (˃100 µg L^−1^) inhibited the growth of *S. elongatus* [88]. 

Interestingly, both MC-containing (5–10 µg L^−1^) and MC-deficient extracts of *M. aeruginosa* (BCCUSP232 and BCCUSP03, respectively) differently influenced the growth of non-toxic *Microcystis* spp., from a reduction in the growth of *M. wesenbergii* (BCCUSP11) to the stimulation in the growth of *Microcystis panniformis* (BCCUSP200) [89]. The growth of the non-toxic strain of *M. aeruginosa* (BCCUSP03) was inhibited by MC-deficient extracts but stimulated by MC-containing extracts, suggesting the allelopathic involvement of other metabolites other than MCs [89]. Exposure of natural *Planktothrix agardhii* population to the MC-containing extracts of two *Planktothrix agardhii*-predominated bloom samples excluded the bioactive role of MCs, but not the other biogenic compounds in the increased biomass as well as MC and chlorophyll a content of the target species [90]. Several studies concerning the interactions between green algae and MC-producing *Microcystis* reported that in addition to MCs, other secondary metabolites influence the phytoplankton community, explaining growth inhibition observed with exposure to non-toxic strains [91,92]. However, a toxic *M. aeruginosa* strain caused a stronger growth inhibition on *C. vulgaris* than did a non-toxic strain [92]. The same was observed for the growth of *Desmodesmus subspicatus* co-cultured with stationary phase cultures of both toxic (PCC 7806) and non-toxic (PCC 7005) strains of *M. aeruginosa,* i.e., faster and stronger inhibition observed with the toxic strain [91]. It is important to repeat such experiments using log phase grown cells since there are many indications that those respond differently to biotic and abiotic conditions [93]. The stationary phase growth of *D. subspicatus* was also inhibited in co-cultivation with the MC-producing *M. aeruginosa* PCC 7806 and when exposed to the spent medium of *Microcystis* [94]. Filtrates from toxic *M. aeruginosa* induced colony formation in *C. vulgaris*, subject to the initial density of the green alga and the growth phase of *M. aeruginosa*, and with continuous field exposure inhibited the growth of the green alga [95]. This recalls the study of Vardi and co-authors [96], who showed that the interspecies interaction between the dinoflagellate *Peridinium gatunense* and a toxic *Microcystis* strain is density-dependent. The organisms recognize the presence of each other and respond accordingly. Unfortunately, many of the experiments performed on organismal communications lack the cell density dimension and thus, it is not clear how relevant they are to the outdoor conditions. 

Regarding the effect of algae on cyanobacteria, Kearns and Hunter [97] revealed that extracellular compounds from a stationary phase *Chlamydomonas reinhardtii* culture ended the production and accumulation of microcystin in *Microcystis*, whereas the same exudates induced the production of anatoxin in *Dolichospermum flosaquae* (previously *Anabaena flosaquae*). Chen and Guo [98] reported that the growth of *M. aeruginosa* (FACHB-1005) was significantly inhibited by filtrates from a *Tetradesmus* *obliquus* (previously *Scenedesmus* obliquus) culture. The complete lysis of the cells of various *Microcystis* strains exposed to the cell-free spent media from the green alga, which co-occurred with toxic *Microcystis* sp. (Lake Kinneret, Israel), indicated the development of secondary metabolites to assure the green alga’s survival [99]. Identification of such secondary metabolites that inhibit the growth of cyanobacterial blooms could help in their mitigation efforts.

#### 2.1.3. MCs and Zooplankton

When considering the interspecies interactions with zooplankton, particularly *Daphnia*, it is important to note the physical limitations of cyanobacterial cells as a food source on the grazers. Cyanobacterial cells are deficient in sterols and long-chain polyunsaturated fatty acid animals [100,101]. Furthermore, the cell morphology and colony formation through aggregation make cyanobacteria poor sustenance. Filamentous cyanobacteria could also inhibit grazing by clogging filtration apparatus [102,103,104], which has been reported to cause trophic blockage on the natural food linkage between primary producers (algae) and zooplanktivorous fish. Exposure of *Daphnia magna* to the extracts of MC-producing and -deficient strains of *P. agardhii* indicated the negative effects of MC-RR and the other secondary metabolites on the growth rate and reproduction of daphnids [105]. However, the filamentous morphology of *Planktothrix*, deficiency of essential lipids and production of non-ribosomal oligopeptides [106] introduces it as low food quality for *Daphnia*. The study of the growth of *D. magna* feeding on *Planktothrix rubescens* as well as the MC-containing wild-type and MC-deficient mutant of *P. agardhii* indicated sterol limitation and the other secondary metabolites superimposed by toxicity in decreased the growth of daphnids [107].

Co-cultivation of *Daphnia similoides* and *Moina micrura*, which were fed on an increased proportion of a toxic *M. aeruginosa* PCC 7806 (0, 20 and 35%) and *A. pyrenoidosa* diet, resulted in the large-sized *D. similoides* to dominate. Towards the end of the experiments, the biomass and carrying capacities of the two cladocerans were both suppressed. However, *D. similoides* was less affected by the increased ratio of the toxic *Microcystis*, indicating the species-specific responses of zooplankton to cyanobacteria [108]. It has been noted that in experiments involving whole-cell exposure, the toxic effects could be minimized due to restricted ingestion of the toxic cells [109,110,111]. However, cyanotoxins were found to be responsible for the inhibitory effects observed in the aquatic herbivores via experiments with filtrates. For example, MC-containing extracts from blooms prevailing in Colombian reservoirs inhibited the growth and reproduction of *Daphnia* spp. [112] and resulted in cardiotoxic effects on *Daphnia similis* [113]. Exposure of *D. magna* to the extracts of *M. aeruginosa* (MC-producer) and *Dolichospermum spiroides* (previously *Anabaena spiroides*) (ATX-producer) in single and a mixture of the two at environmentally relevant concentrations reduced the feeding activity and survival of the daphnids [114]. Furthermore, the combined two toxins inhibited the feeding rates of *D. magna* more potently than exposure to the individual toxins. Experiments with mixed toxins or rather bloom extracts or culture filtrates would provide better insight into the allelochemical driven interactions in aquatic ecosystems as cyanobacterial toxins do not exist in single but rather in a complex matrix of other secondary metabolites which could act either synergistically or antagonistically.

As is the case with bacterial and algal interaction with the toxic cyanobacteria, studies have excluded the allelopathic role of MCs, suggesting the involvement of other secondary metabolites in the interspecies interactions. Acute and chronic exposure of *D. magna* to MC-containing and non-containing blooms extracts at ecologically relevant concentrations excluded MCs’ involvement in the reduced reproductive health of daphnids [115], and thus their role as grazing deterrent is questionable. High concentrations of MCs also did not affect the abundance of zooplankton *Daphnia* in super-eutrophic ponds in Alabama and Michigan (U.S.) [116]. Interactions between *Daphnia* and *M. aeruginosa* PCC 7806 indicated that inducible defense mechanisms included the elevated production of toxic compounds other than MCs such as aerucyclamide B and D, cyanopoeptolin B and microcyclamide 7806A in the cyanobacterium [117]. Another example came from studies in Rio Grande do Norte, Brazil, where the biomass of calanoid copepods was positively correlated to MC concentration. However, MC (0.13–5.26 μg L^−1^) produced mainly by *Microcystis* spp. did not have any significant influence on the reduction of microzooplankton biomass (protozoans and rotifers) in eutrophic reservoirs, possibly due to the establishment of MC-resistant genera [118]. 

Exposure of *Brachionus calyciflorus* to both toxic and non-toxic strains of *M. aeruginosa* decreased the population growth rate of the rotifer. The rotifer was more sensitive to the toxic *Microcystis* than the non-toxic strain since the higher proportion of toxic *Microcystis* induced a significantly higher mortality rate [119]. Liang et al. [120] reported that the increased density of *M. aeruginosa* PCC 7806 caused more significant toxicity in *B. calyciflorus*, suggesting that the release of MCs from *Microcystis* due to rotifer grazing negatively affected both the growth and reproduction of the rotifer. 

According to the previous reports, abiotic environmental factors influenced the toxicity of MC-LR. Nandini et al. [121] showed that the survivorship and reproduction of the rotifer *B. calyciflorus* exposed to extracts of a *Microcystis* bloom from Valle de Bravo reservoir (Mexico) were improved at a temperature of 25 °C compared to 20 °C. Liang et al. [122] found that the exposure of *B. calyciflorus* Pallas to low doses of MC-LR (10 and 30 µg L^−1^) and nitrite (1 and 3 mg L^−1^) synergistically improved the lifespan and reproductive rate of the rotifer. In contrast, a higher dose of MC-LR and nitrite (100 and 5 mg L^−1^, respectively) reduced the lifespan and reproductive performance at 20 °C and 25 °C. By increasing the temperature to 30 °C, the toxic effects of toxicants on the rotifer were strengthened, i.e., increased reactive oxygen species (ROS) and decreased heat-shock proteins (HSP) gene expression. These experiments highlight the importance of mesocosm studies, which take into account abiotic factors.

Generalizations regarding the interspecies interactions between cyanobacteria and zooplankton have been challenging due to the varied responses of the same species, strains and even clones of the same species [111,123,124,125,126], raising the possibility that different cell densities were used in various experiments, besides the probable involvement of the other secondary metabolites [117]. The increased tolerance may also be attributed to the history of the cells used in the competition experiments or even exposure in previous generations [110,111]. Exposure periods used in laboratory experiments are short relative to bloom events. Considering the short generation time of zooplankton, adaptation to cyanobacterial toxins in nature is likely [127] and should be considered in these investigations. Understanding the negative effect of allopathic chemicals from cyanobacterial on zooplankton is essential to avoid impacts on energy transfer to higher trophic levels and disturb the natural food web during blooms [128].

#### 2.1.4. MCs and Aquatic Macrophytes

When aquatic macrophytes were exposed to MC-producing cyanobacteria as well as MC as a pure toxin, the growth and photosynthesis were significantly inhibited. MC-producing *M. aeruginosa* induced an increase in the activities of the antioxidative enzymes and caused lipid peroxidation in *Egeria densa*. Prolonged exposure resulted in growth reduction and inhibition of root and shoot emission in the aquatic plant [129]. In the same macrophyte, exposure to a low *M. aeruginosa* cell density (OD_730_, 0.04) caused oxidative stress and negatively influenced the pigments and chlorophyll-a content [130]. Exposure of *Vallisneria natans* to MC-LR (1.129 μg L^−1^) released from *M. aeruginosa* at the death phase induced oxidative stress in the macrophyte. The MC-LR exposure also caused an alteration in the abundance and structure of the microbial community in biofilms present on the leaves of the macrophyte, and increased variations were seen in extracellular polymeric substances (EPS) of these periphyton biofilms [131]. Thus, MC exposure not only directly affects macrophytes but also could abolish symbiotic relationships with microbes present in leaf biofilms, which could provide essential metabolites to the macrophytes, further affecting the plants. A root exudate produced by a floating macrophyte *Eichhornia crassipes*, water hyacinth, was reported to increase the allelopathic effects of *M. aeruginosa* on the green alga *Scenedesmus quadricauda* via stimulating the release of allelochemicals from the cyanobacterium [132]. 

Exposing *Ceratophyllum demersum* to MC-LR at an environmentally relevant concentration of 5 µg L^−1^ caused oxidative stress and inhibited growth and photosynthesis [31,133]. However, in *Typha angustifolia* Linn, MC-LR at a similar environmental concentration (4.6 μg L^−1^) stimulated photosynthesis, based on enhanced Rubisco activity and an increased net photosynthetic rate after six weeks of exposure [134]. In *Iris pseudacorus* L. seedlings exposed to MC-LR (50, 100, 250 and 500 μg L^−1^), oxidative stress was induced and growth was inhibited. Furthermore, at an MC-LR concentration of 100 µg L^−1^, the nitrate uptake was increased, which promoted the alkalinity of the water and inhibited the growth of the roots [135].

**Figure 2:**
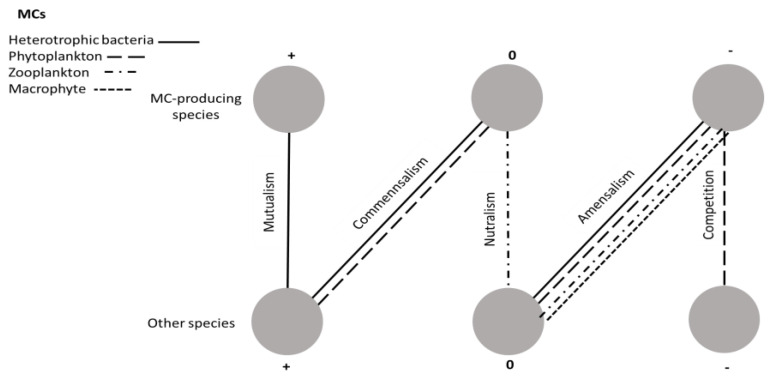
Interspecies interactions between microcystin producers and other co-occurring organisms in aquatic ecosystems. Lines indicate the interaction between microcystin and heterotrophic bacteria, phytoplankton, zooplankton and macrophytes.

The interspecies interactions between MC producers and the other aquatic community members are difficult to characterize in general terms (Figure 2) as mutualism is observed in terms of nutrient cycling and competition for nutrients. In general, MC seems to have inhibitory effects on the biodiversity and abundance of aquatic organisms (amensalism), which could be interpreted as lending a competitive advantage to the producer. To further complicate understanding the complexity of the interactions, many studies have been restricted to understanding the effects of one species on another and no information was gathered on the interaction in the opposite direction. Additionally, prolonged exposure to MC may raise localized adaptation to the hepatotoxin.

### 2.2. Anatoxins (ATXs)

The anatoxin (ATX) group, which are potent bicyclic alkaloid neurotoxins, consists of anatoxin-a, homoanatoxin-a and anatoxin-a(s). ATX-a, mainly produced by *Dolichospermum* sp. (*Anabaena*) and *Aphanizomenon* sp., binds to nicotinic receptors of motor neurons, continuously stimulates the muscles and causes muscles failure in the respiratory and cardiovascular systems that can lead to death (as reviewed by Christensen et al. [136]). 

#### 2.2.1. ATXs and Heterotrophic Bacteria

The diversity of the bacterial community assemblage associated with benthic neurotoxin-producer dominated mats has been reported in recent studies. In the Eel River (Northern California, U.S.), the microbial assemblage within benthic ATX-producing and -deficient *Phormidium* mats were found to differ substantially [137]. The microbial community related to ATX-producing species was dominated by Bacteroidetes, Proteobacteria and Verrucomicrobia. They contribute to ATX degradation as well as carbon, nitrogen and sulfur cycling that facilitated the growth of cyanobacteria. The toxic mats contained a lower abundance of Burkholderiales, suggesting that the adverse effects on these family members were due to ATX-a exposure. These toxic mats contained a higher abundance of Sphingomonadales, an order with known MC-degrading species. However, MC and ATX are structurally different, and distinct genes may be involved in ATX degradation, which should be a priority of future research. The variation in the microbial community (dominated by Bacteroidetes and Proteobacteria) within ATX-producing *Microcoleus autumnale* (basionym *Phormidium autumnale*) -dominated mats was also reported in two New Zealand streams concerning the variability in ATX concentration. However, the presence of various co-inhabiting organisms such as diatoms and eukaryotic algae in the mats, as well as the environmental and micro-environmental conditions, may influence the bacterial community structure as well [138]. Research regarding the effect of ATXs on the bacterial assemblage in aquatic ecosystems is still at its early stages. In efforts going forward, not only the effects of this neurotoxin on the heterotrophic bacterial assemblage need to be studied but also, if community structural shifts and changes occur, what would be the implications of these for the ATX-producing species.

The co-occurrence of MCs and ATX-a in lakes and river networks [139,140] has raised a need for mixed toxins investigations. Li et al. [141] indicated that exposure to ATX-a and MC-LR individually and combined at environmentally relevant concentrations (0.05–5.00 μg L^−1^) changed the microbial diversity and abundance in *V. natans* leaf biofilms. In treatments with MC, Proteobacteria was commonly observed and in ATX-a, and combined toxins exposures, Actinobacteria, Cyanobacteria and Planctomycetes were commonly detected. Moreover, the increased concentration of AHL (N-acyl-homoserine lactones), known as a quorum-sensing signaling molecule in gram-negative bacteria, indicated the effects of cyanotoxins on the quorum sensing-directed behaviors such as regulation of the formation of biofilm and its microbial community constructions in the periphyton [142]. Experiments including bloom material extracts would further deepen the understanding of the interspecies effects of a cyanobacterial bloom considering the effects of other toxins (such as endotoxins and BMAA) and secondary metabolites that play a role. 

#### 2.2.2. ATXs and Phytoplankton

The growth of *M. aeruginosa* BCCUSP 232, exposed to ATX-a (5, 10, 25 and 50 μg L^−1^), was not altered, possibly explaining the co-occurrence of MC- and ATX-producing species in multispecies cyanobacterial blooms. However, the total protein, chlorophyll-a and MC content were reduced, and oxidative stress was promoted in *M. aeruginosa* as a function of increasing concentrations of ATX-a [143]. Chia et al. [144] reported that ATX-a (6.25 and 25 μg L^−1^) influenced the physiology of *M. aeruginosa* BCCUSP 232 and *Acutodesmus* *acuminatus* (Chlorophyta) differently under various light and nitrogen conditions. Exposure to ATX-a under various light (optimum light: 30 μmol m^−2^ s^−1^ for *M. aeruginosa* and 40 μmol m^−2^ s^−1^ for *A. acuminatus*. and low light 10 μmol m^−2^ s^−1^) and nitrogen (optimum and high nitrogen concentrations, 1.8 and 9 mM, respectively) regimes did not alter the growth of *M. aeruginosa* while the growth of *A. acuminatus* was significantly reduced under optimum and high nitrogen concentrations. Exposure to ATX-a has also reduced the pigment and MC content of *M. aeruginosa* regardless of light conditions. Nutrient-dependent allelopathic interactions between *Microcystis* and *Dolichospermum* (previously *Anabaena*) indicated that the MC-producing *Microcystis* dominated under high nitrogen and low phosphorous while *Dolichospermum* dominated under low nitrogen levels [32]. These findings emphasized the need to take the combined effects of environmental conditions and cyanotoxins in ecotoxicological investigations into consideration. 

Chia et al. [145] studied the effects of the cyanotoxins ATX-a and MC-LR as single toxins and combinations of both (25 µg L^−1^ each) on *Microcystis* spp., *T. variabilis* and the green alga *Selenastrum capricornutum*. Following exposure to the cyanotoxins, ATX-a or MC-LR, the growth of *Microcystis* LE-3 was inhibited. ATX-a (individually and combined with MC-LR) inhibited nitrogen fixation in *T.* *variabilis* UTEX B377. The combined cyanotoxins promoted the growth of green alga while causing a synergistic decrease in the photosynthesis efficiency in *Microcystis* LE-3 and *T. variabilis* UTEX B377. In addition, the combined treatments resulted in the increased intracellular level of MCs in *Microcystis* LE-3, suggesting the protective role of MCs against the induced oxidative stress. These data demonstrated the diverse effects of cyanotoxins on the growth and physiology of phytoplankton species, which may explain the succession of the green algae following the collapse of toxic cyanobacterial blooms. Even though species-specific effects related to the cyanotoxins individually and combined were observed, the effects of the combined toxins on some strains were greater than with single toxin exposures [145]. 

A mesocosm study has shown that *D. flosaquae*, which can produce ATX-a and MC-LR, outcompeted the *C. reinhardtii* by inducing the green alga to settle, creating a free zone for the cyanobacterium [146]. More studies pertaining to the morphological and physiological effect of the ATX on green algae are required to understand their role, if any, as an allelochemical and in the succession of ATX-producing cyanobacteria. 

Concerning the co-occurrence of various cyanotoxins such as MC and ATX, investigation of the synergic association of cyanotoxins and environmental factors may clarify the factors regulating the bloom dynamics towards the dominance of one species. Extended laboratory and field research into the effect of ATXs on other cyanobacteria as well as the producing strain may give insight into its role as a communication molecule. Repeating experiments with both ATX-a and ATX-a(s) may, such as with the isoforms of MC, reveal varied effects.

#### 2.2.3. ATXs and Zooplankton

Abreu and Ferrão Filho [147] reported that both the intact cells and aqueous extracts of ATX-producing *D. spiroides* (ITEP-024) caused reduced survivorship and growth rates in *D. similis*. The greater effects of intact cells compared to the aqueous extracts suggested more effective uptake of toxin via the digestion of the intact cells in daphnia’s gut. Exposure of *D. magna* to ATX (0.5–50 μg mL^−1^) resulted in decreased swimming speed, abdominal claw movements and heart rate [148]. 

Extracts of the toxin-producing *P. agardhii* and *Dolichospermum* spp. bloom scums from different lakes in Poland had a greater adverse effect on the survivorship of the freshwater zooplankton *B. calyciflorus* and *Daphnia pulex* compared to pure MC-LR and ATX, suggesting the potential contribution of the other secondary metabolites to the overall toxicity of cyanobacteria on invertebrates. Moreover, the toxicity was species-specific, as daphnids were more sensitive than rotifers to pure ATX and MC-LR [25]. As with MCs, studies considering the tolerance evolution of zooplankton with prolonged exposure are lacking to understand the interspecies interaction development with time during a bloom event.

The recent study by Schwarzenberger and Martin-Creuzburg [149] indicated that feeding several clones of *D. magna* with ATX-a producing *Tychonema bourellyi* (undiluted by other feed) resulted in a reduced growth rate and increased expression of nicotine-acetylcholine receptors (NAR) gene. In contrast, feeding with 50% of *T. bourellyi* caused no reduction in the growth rate. Only in one clone was the NAR gene expression increased. The offspring fed with 50% *T. bourellyi* showed an increased growth rate, suggesting the maternal transfer of the increased gene expression to offspring as an adaptive response to increase its fitness.

#### 2.2.4. ATXs and Aquatic Macrophytes

ATX-a (15 µg L^−1^) inhibited the growth, reduced the chlorophyll content, and induced oxidative stress in aquatic in macrophyte *C. demersum* [150]. Exposure of *C. demersum* to the lower concentrations of ATX (≤5 μg L^−1^) elevated the tocopherol contents, while a higher concentration (50 µg L^−1^) reduced tocopherols and increased lipid peroxidation in the submerged plant [151]. ATX-a and MC-LR as single and mixed cyanotoxins at environmentally relevant concentrations (0.05–5.00 μg L^−1^) induced oxidative stress in the submerged macrophyte *V. natans* as well [141]. These data give us an understanding of the physiological implications of toxin exposure; however, the effects at the population level are still lacking. It is unclear whether macrophyte species are affected to such an extent that entire populations decline and perish. 

Considering all the data presented, the presence of ATX has negatively influenced the phyto- and zooplankton species as well as aquatic plants while changing the microbial community dynamics towards the dominance of ATX-degrading assemblages. The co-occurring cyanotoxins, MC and ATX, despite their different modes of action, had synergic interactive effects on the aquatic biota, which should gain more attention in future studies.

### 2.3. Saxitoxins (STXs)

Saxitoxins (STXs), also commonly referred to as paralytic shellfish toxins (PSTs), are a group of natural neurotoxic heterocyclic guanidinium compounds with sodium channel blocking action in humans and marine mammals [152,153,154] as well as cyanobacteria; however, to a lesser extent than their influence on the eukaryotic membrane channels [155]. STXs are produced by certain marine dinoflagellates, such as *Alexandrium* spp. and *Gymnodinium* spp., as well as some cyanobacteria, e.g., *D. circinale*, *A. flos-aquae*, *R.* *raciborskii*, *Microseira wollei* (previously *Lyngbya wollei*) and *Planktothrix* spp. [136,156]. To date, no information exists on the effects of cyanobacterial produced STXs on macrophytes in freshwater ecosystems. 

#### 2.3.1. STXs and Heterotrophic Bacteria

Even though marine STX-producing dinoflagellates are well studied, those in freshwater environments remain elusive, and data on cyanobacterial produced STX is lacking. Several symbiotic bacterial phyla (dominated with Proteobacteria) were identified in association with the host STX-producing dinoflagellate *Alexandrium tamarense* [157]. In the Indian River Lagoon, Florida, the bacterial species of *Arcobacter* sp. and *Truepera* sp. had the maximized correlation with STX in the dinoflagellate *Pyrodinium bahamense* bloom [158]. 

Raudonis [159] reported that the a-Proteobacteria dominated the bacterial community associated with Australian freshwater PST-producing strains of *D. circinale*, which mainly produced C-toxins (Cl and C2), a lower amount of GTXs (GTX 2 and GTX 3) as well as trace amounts of STX and other GTXs. Significant knowledge gaps remain regarding the probable associated microbial assemblage related to the freshwater cyanobacteria STXs.

#### 2.3.2. STXs and Phytoplankton

The inhibitory effects of STX on phytoplankton species were reported by Do Carmo Bittencourt-Oliveira et al. [160]. Following exposure to both STX-containing (0.5–10 μg L^−1^) and STX-deficient extracts of *R. raciborskii*, the growth of a toxic *M. aeruginosa* strain and non-MC producing strains of *M. wesenbergii*, as well as the green algae *Tetradesmus lagerheimii* (previously *Scenedesmus acuminatus*) was inhibited. However, the inhibitory effects of the STX-containing extracts were greater than the non-STX extracts and were positively related to the concentration of STX. Both extracts enhanced the total MC content of toxic *M. aeruginosa* [160]. Since the purified STX (0.75–48 μg L^−1^) had no growth inhibitory effect on the growth of *C. reinhardtii* [161], it would be of great value to better characterize the chemical composition of the crude extracts to clarify the potential contribution of the other bioactive compounds that may influence the observed toxicity. Exposure of *C. reinhardtii* to different concentrations of purified STX (0.15–1.1 μg L^−1^) induced the activities of the antioxidative enzymes in the green alga at the highest exposure concentration [162]. 

#### 2.3.3. STXs and Zooplankton

Haney et al. [163] reported that exposure of *Daphnia carinata* to the filtrates of *A. flos-aquae* and purified STX (2, 200 and 2000 μg L^−1^) increased the rejection rate of particles by the post-abdomen and reduced the thoracic appendages beating rate of daphnia, suggesting that the toxin can act as a deterrent. Exposure of *D. similis* to intact cells of STX-producing *R. raciborskii* (250–500 μg L^−^^1^) inhibited the swimming ability as well as feeding behaviour (movements of antennae and thoracic limbs) and decreased the heart rate of the daphnids [164]. The living cells (0.5 mg L^−1^) of the toxic strain of *R. raciborskii* CYLCAM-2 (isolated from the Camorim reservoir (Rio de Janeiro, Brazil)) caused a decrease in the somatic growth and reproduction of the cladocerans, *Daphnia* and *Ceriodaphnia*. The inhibitory effects were greater with an increased proportion of the cyanobacterium (˃50%) in the food mixture containing the green algae [165]. As there were no nutritional limitations between the two exposure scenarios, this suggested the toxic effects of the cyanobacterium. In contrast, in a mesocosm study, *Daphnia laevis* (20 individuals L^−1^) reduced the biomass of STXs-producing *R. raciborskii* CYLCAM-2, possibly via direct ingestion or facilitation of its ingestion by other small zooplankton such as rotifers [166]. 

Calanoid copepod *Eudiaptomus gracilis* consumed fewer of the STX-producing (STX+) *R. raciborskii* strain cells compared to the STX-deficient strain (STX-), suggesting the involvement of STX against the grazing pressure of zooplankton [167]. In contrast, the prolonged exposure to cyanotoxin producers caused a selective pressure on shifting the zooplankton community towards more adapted genotypes within a species. An acute immobility test with exposure to fresh biomass of STX-producing *R. raciborskii* (cell biomass: 150, 300 and 500 μg L^−1^) showed that the zooplankton *Macrothrix spinosa* isolated from the previously bloom-recorded lake (Pernambuco, Brazil) was more tolerant than a cladoceran from the bloom-free area, suggesting the evolution of toxic tolerance in subsequent generations [168]. 

Exposure of *D. laevis*, *D. similis* and *M. micrura* to the single and combined diet of MC-producing strains of *M. aeruginosa* and STX-producing strain of *R. raciborskii* caused variable responses in different cladoceran species in terms of survival, clearance rates, mobility and population growth rates [169], also indicating the negative synergistic effects of the studied cyanotoxins. These studies with toxin mixtures are important to understand the mixed effect and should also consider non-toxic effects by including bloom extract exposure in addition to pure toxins in single.

Collectively, STX-producers benefitted from toxin production, which inhibited the growth of phytoplankton species and served as a defence mechanism against the grazing force of zooplankton. However, most STXs related investigations have been made on marine ecosystems. To our knowledge, few studies have focused on the effects of STXs on freshwater biota, as detailed above. Further studies, including a larger diversity of organisms, would benefit the overall understanding of the role of STX in ecosystems driving species interactions.

### 2.4. Cylindrospermopsin (CYN)

Cylindrospermopsin (CYN), a selective cytotoxin, is an inhibitor of protein synthesis, which is produced by planktonic cyanobacteria, e.g., *R. raciborskii*, *Umezakia natans*, *Aphanizomenon ovalisporum*, *Anabaena bergii, Raphidiopsis curvata*, *A. flos-aquae*, *Anabaena lapponica* and *M.* *wollei*, which are generally found in tropical and subtropical lakes [170,171]. It has been reported that CYN affects aquatic biota, acts as an allelochemical in cyanobacteria together with other species assemblages and potentially contributed to the success of the producing organisms (as reviewed by Rzymski and Poniedziałek [172]). Of all the toxins discussed here, CYN is the only cyanotoxin known to exist extracellularly during a bloom event, and it would be of value to understand if this holds any significance to interspecies interactions of CYN producing cyanobacteria. 

#### 2.4.1. CYN and Heterotrophic Bacteria

Rasmussen et al. [173] reported the minimal inhibitory concentration of 300 µg L^−1^ for CYN, extracted from *R. raciborskii*, against both gram-negative and gram-positive bacteria *E. coli*, *B. subtilis*, *Staphylococcus aureus* and *Pseudomonas aeruginosa*. These findings suggested that CYN was not a potent antibacterial agent at an environmentally relevant concentration.

Concerning CYN-biodegradation, Wormer et al. [174] reported that bacterial community, naturally co-occurring with cyanobacterial blooms containing CYN-producing *A. ovalisporum*, were not able to degrade CYN during the 40-day period; however, recent studies indicated that several bacterial species such as *Aeromonas* sp. (isolated from Rusałka Lake, Poland) [175] as well as *Pseudomonas* sp. *Ideonella* sp. and Comamonadaceae [176] were capable of CYN-biodegradation in CYN-removal studies. 

#### 2.4.2. CYN and Phytoplankton

CYN-producing *A. ovalisporum* inhibited the benthic *Chlorococcum* sp. green alga [177]. In mixed culture treatments (green alga to cyanobacterium ratios: 1:1, 1:2 and 1:4), a higher initial cell number of the cyanobacterium had a more significant inhibitory effect on the growth of green alga. CYN-containing extracts of *A. ovalisporum* inhibited the growth of the green alga *Chlorococcum* sp. concentration-dependently, where the inhibitory effects increased with the increasing concentration of the crude extract. Changes in the nutrient content in the presence of the cyanobacterium in mixed cultures were not detected, refuting the possibility of resource competition and emphasized the allelopathic inhibitory role of CYN. However, other bioactive compounds present in the cells or extracts may have played a role; but this was not investigated in these studies [177]. 

The CYN- and STX-containing filtrates from a *R. raciborskii* LEGE 99043 culture inhibited the growth of green alga *Ankistrodesmus falcatus*. The allelopathic inhibition was enhanced under high light intensity and temperature, as well as phosphorous-limited conditions as STX and CYN production was found to be a function of light and temperature [178]. 

In contrast, Pinheiro et al. [179] showed that MC-LR and CYN at the environmentally relevant concentrations did not influence the growth of the microalgae *Nannochloropsisn* sp., *C. reinhardtii* and *C. vulgaris*. Neither toxic (CYN-producing) nor non-toxic strains of cyanobacteria affected the photosynthesis of *S. quadricauda* [180]. Kovács, Tóth and Pálffy [180] also tested several other toxin-producing (MC and ATX) species and found no adverse effects on photosynthesis compared to non-toxic strains.

Further studies showed the growth of MC-producing *M. aeruginosa* Kütz. (Zapomelova 2006/2) was inversely related to the CYN concentration it was exposed to; lower concentrations (1 and 5 μg L^−1^) slightly inhibited, and the higher concentrations (10 and 50 μg L^−1^) strongly inhibited growth and induced cell necrosis. MC production was significantly decreased at all concentrations of CYN tested. However, exposure to cell-free filtrates of non-CYN producing *R. raciborskii* inhibited the growth and MC production of *M. aeruginosa* as well, suggesting that the non-CYN producing strain possibly produces metabolites that mimic the inhibitory action of CYN [30]. The exudates of the non-toxic strain *R. raciborskii* CYRF-01 in mono- and co-cultures with *M. aeruginosa* induced colony formation in the coexisting *Microcystis*. Moreover, the growth of *M. aeruginosa* was inhibited when exposed to filtrates of mixed cultures with a high proposition of *R. raciborskii* (25–75%), suggesting the ability of non-CYN-producing *R. raciborskii* to produce allelochemicals [181]. Of immense value to understanding the interspecies interaction of toxin-producing strains would be the chemical characterization of all secondary metabolites produced and understanding their synergistic relations.

Chia et al. [182] found that the allelopathic capability of CYN varied in relation to the changing light and nitrogen conditions, emphasizing the significance of physiochemical conditions, also highlighting the value of mesocosm experiments in this regard. Following exposure to CYN (6.25 and 25 µg L^−1^), the total MC content of *M. aeruginosa* was increased under limited light (10 μmol m^−2^ s^−^^1^) after 24 h, while it was reduced under optimum light (30 μmol m^−2^ s^−1^) as well as optimum and limited nitrogen (NaNO_3_; 1.8 and 0.04 mM, respectively) after 120 h. The presence of CYN inhibited the growth of *M. aeruginosa* regardless of the level of light intensity and nitrogen conditions. Conversely, the growth of *A. acuminatus* (Chlorophyta) was inhibited under low light and limited nitrogen, regardless of the presence of CYN. However, in both exposed species, CYN induced oxidative stress, which was amplified under the limited light and nitrogen.

The biological role of CYN was revealed in a set of experiments where the CYN producing *A. ovalisporum* was allowed to interact with other organisms [183]. For example, exposure of *C. reinhardti* to either the filtrates from the CYN-producing *A. ovalisporum* (isolated from Lake Kinneret, Israel) culture or purified CYN induced the activation of the PHO regulon and secretion of alkaline phosphatase (APase) from the green alga. This was despite the fact that the algal media was phosphate-rich. In mixed cultures, enzyme-labelled fluorescence (ELF-APase) signals were detected in *Chlamydomonas* and a *Debarya* sp. but not in *A. ovalisporum,* where the activation of the PHO regulon and CYN production were strongly induced under phosphate limiting conditions. It was concluded [183] that the CYN production under low phosphorus serves to enhance phosphorus availability in the media following cleavage from dissolved organic substances with the aid of APase excreted by various algae. The high-affinity Pst system in *A. ovalisporum* enables it to outcompete others for the available phosphorus. It appears as if CYN producing strains enslave the other phytoplankton species that increases the external inorganic phosphorus supply, thereby facilitating the growth of the toxic strain [183]. It is suggested that the ability to fix atmospheric N_2_ by CYN producing strains may provide them with an advantage over others in nutrient-limited water bodies. Exposure of *Scenedesmus obtusus* to the extracts of CYN-producing and -deficient strains of *Aphanizomenon*, as well as CYN-deficient extracts supplemented with CYN, showed that only the extracts of the toxic strain-induced alkaline and acidic phosphatases activity in the green alga. These data indicate the presence of other metabolites with similar effects that are absent in the toxin-deficient strain or not contributed to CYN toxicity [184]. 

#### 2.4.3. CYN and Zooplankton

Toxic *R. raciborskii* has been shown to shift the structure of the zooplankton community in the St. Johns River System, Florida [185]. At a lower density of *R. raciborskii*, the zooplankton was more diverse and contained larger species, dominated by rotifers. In contrast, the higher density of *R. raciborskii* accompanied by the greater number of smaller zooplankton, which may reduce the grazing pressure of zooplankton on *R. raciborskii* due to the size-selection of smaller algae by microzooplankton species.

Concerning the effects of CYN on zooplankton, CYN-producing *R. raciborskii* caused a heftier obstructive effect on the fitness and growth of juvenile *D. magna* compared to the non-toxic strain, suggesting the partial contribution of CYN in the overall toxicity of producing strain [186]. This idea was further emphasized in another study by Nogueira et al. [187], where the growth and survivorship of *D. magna* were reduced by feeding on two CYN- producing *R. raciborskii* and *A. ovalisporum*. However, only *R. raciborskii* caused dissociation of epithelial cells in the midgut and diverticula. The physiological (induced oxidative stress) and various behavioral changes (mobility) that were observed following the exposure of *D. magna* to the extracts and living cells of non-MC and non-CYN cyanobacterial strains empowered the probable presence of other bioactive toxic compounds [188].

#### 2.4.4. CYN and Macrophytes

Studies on the effects of CYN on aquatic macrophytes have centered on understanding the physiological effects of exposure, such as chlorophyll content, oxidative stress and in some instances, growth. In a study by Flores-Rojas et al. [189], exposure to pure CYN caused growth inhibition in the floating macrophyte *Lemna minor* L. when exposed to the environmental concentrations of (0.25 and 2.5 μg L^−1^) and induced oxidative stress (with 2.5 and 25 μg L^−1^) but was quickly recovered by the macrophyte’s sufficient antioxidative defense system [190]. In *E. densa*, CYN (pure toxin in single, 2.5 and 25 µg L^−1^) exposure stimulated growth for the first two weeks, but thereafter growth and biomass significantly decreased compared to controls. The antioxidative enzyme activities increased, and the pigment concentrations decreased with exposure but normalized after seven days [191]. The authors reported that even though chlorosis was observed during the exposure period of 32 days with the highest exposure concentration of 25 µg L^−1^, the plants remained alive and new leaves formed. As CYN is stable under a range of light, heat and pH conditions [192], long term exposure experiments are essential in determining the longstanding effects of exposure and identifying organisms’ adaption and detoxification mechanisms that allow them to co-occur with toxic blooms.

Exposure of *Azolla filiculoides* to CYN-containing extracts (0.05, 0.5 and 5 μg mL^−1^) from *A. ovalisporum* showed that CYN at the highest level (5 μg mL^−1^) inhibited the growth of the aquatic fern. At the same time, the protein content, chlorophyll, carotenoids and the antioxidative enzymes (glutathione reductase (GR) and glutathione-S-transferase (GST)) activities increased [193]. The CYN-containing crude extract of *A. ovalisporum* (BGSD-423) caused more significant unfavorable effects than pure CYN (each at 20 µg mL^−1^) on the growth of the aquatic plants *L. minor* L. and *Wolffia arrhiza* [194].

The results from previous studies regarding the effects of CYN in aquatic plants vary and seem to depend on the CYN concentration, which could either inhibit or stimulate growth. Based on the fact that the extracts caused exacerbated effects, CYN toxicity is reinforced by synergism with other secondary metabolites. Ultimately, CYN-producing species utilize the advantages of toxin production to outcompete the other phytoplankton species depending on the environmental conditions, as well as zooplankton and aquatic plants. However, some organisms may have adaptive mechanisms to assure their survival and co-occurrence in the aquatic ecosystems. 

### 2.5. BMAA

β-N-methylamino-L-alanine (BMAA) is a non-proteinogenic amino acid that can naturally occur in a soluble as well as protein-associated form. The neurotoxin, which is produced in response to nitrogen starvation [195], is produced by the majority of cyanobacterial species [196,197] as well as diatoms [198,199] and dinoflagellates [200]. 

#### 2.5.1. BMAA and Heterotrophic Bacteria

In 2011, Visser [201] showed that BMAA was taken up on exposure but was not cytotoxic to the yeast *Saccharomyces cerevisiae* or the bacterium *E. coli*. Van Onselen et al. [202] later confirmed that even though taken up, BMAA seems to be non-toxic to a variety of prokaryotes, as it may not incorporate into bacterial proteins but only bound to the surface. Therefore, the available information suggests that BMAA has no role in competition with the prokaryotic assemblage in aquatic ecosystems. 

#### 2.5.2. BMAA and Phytoplankton

Previous studies report that cyanobacteria take up BMAA from their environment [203,204]. BMAA absorption by *Nostoc* sp. PCC 7120 resulted in the inhibition of nitrogen fixation and growth arrest as well as accumulation of glycogen. In heterocystous, diazotrophic cyanobacteria, BMAA inhibited heterocyst formation when nitrogen was limited [205]. In *Synechocystis* PCC 6803, the internalized BMAA was transaminated by glutamine oxoglutarate aminotransferase [204]. The current data suggest that BMAA plays a role in signally among cyanobacteria. Information regarding the effects on the eukaryotic algae is lacking, and thus a conclusion cannot be drawn as to whether signaling is limited to cyanobacterial only.

#### 2.5.3. BMAA and Zooplankton

Lürling et al. [206] acutely exposed *D. magna* to pure dissolved BMAA and found that even at the highest concentration (45% mortality with 10 mg L^−1^), BMAA was not lethal to the daphnids but significantly affected the mobility and reproduction. Faassen et al. [207] later confirmed these findings and reported that offspring from pre-exposed daphnids expired sooner than those from unexposed mothers. Esterhuizen-Londt et al. [208] found that after a 24 h period of exposure to the pure dissolved toxin, BMAA was taken up by *D. magna*; however, it was not bioconcentrated, nor was it detected in the protein-associated form. Inhibited activities of the antioxidative enzymes were associated with the internalization of BMAA by the daphnids. Interestingly, BMAA was found intracellularly when fed with BMAA-producing cyanobacterium. This data confirms that BMAA is not involved as a toxic molecule to aid in competition during bloom establishment.

#### 2.5.4. BMAA and Macrophytes and Macroalgae

BMAA was previously detected in macrophytes collected from reservoirs in Nebraska [209]. In laboratory experiments, the macrophyte *C. demersum* rapidly took up free dissolved BMAA from its surrounding, of which a portion became protein-associated intracellularly [210]. The internalized BMAA caused restraint on the typical functionality of the oxidative defense enzymes in the macrophyte [211]. *C. demersum* did not metabolize the cellular BMAA by catabolism but rather regulated via covalent modification followed by sequestration [212]. As BMAA inhibits the antioxidative enzymes in exposed organisms and is said to be produced by the majority of cyanobacterial tested globally as well as diatoms and dinoflagellates, BMAA could abet the toxicity of other toxins produced by the blooming cyanobacterium. As MC, ATX and CYN exposure is associated with oxidative stress and the exposed organism overcomes the adverse effects via the antioxidative system, BMAA, which inhibits these enzymes, could impede the organism from recovering. Thus, experiments including toxin mixtures and extracts are important to understand the overall interaction of allelochemicals during blooms. The study by Contardo-Jara et al. [213] gave a brief insight into this possibility. The green macroalga *Aegagropila linnaei* took up more BMAA in the presence of MC-LR than single toxin exposure. In addition, exposure to a combination of the two toxins elicited enhanced oxidative stress in the alga compared to exposure to the toxins in single [213]. 

The role of BMAA within interspecies interactions, if any, is still poorly understood, with no definite benefits given to the producer in terms of a competitive advantage within its environment. Perhaps BMAA is merely a by-product of nitrogen storage metabolism with coincidental neurotoxic properties; nevertheless, this remains controversial.

## 3. Concluding Remarks

Coevolution, strong flexibility and adaptability have affected the antagonistic interactions between the co-occurring species [181,214]. The theory of biodiversity states that niche differentiation assists coexistence by weakening competition [203,204]. Phylogenetic niche conservatism will lead to using the same resources and, therefore, a stronger competition between species. However, as seen in the above compendium, some of the studies have reported conflicting results regarding the allelopathic effects of cyanobacterial toxins at environmental concentrations and their role in interspecies interactions [179]. The questions remaining are whether these conflicting interactions are based and triggered by environmental issues, nutrient availability, growth phase or even cell-cell signaling. It is also unclear whether phenotypic variations control species interactions and therefore trigger coexistence. If phenotypic variations are the main influencer, then that would mean this arises from the gene expression level. Many of the effects of cyanotoxins were strain-dependent; or possibly even specific to laboratory conditions. Thus, strain-specificity in interspecies interactions must always be considered [180,181], and investigations under environmental conditions in mesocosms experiments or lake monitoring would be more insightful. Phenotypic variations developing under laboratory culture conditions, or diverse environmental conditions, may contribute as well. Considering the difficulty in differentiating between the responses to toxins and the consequence of environmental factors such as temperature, light and nutrient availability [32,92], a combination of the two should be explored and included in laboratory set-ups, including mesocosm studies. Some species, such as archaea, were detected under natural environmental conditions in field samples but absent in laboratory and field experimental set-ups [215]. Combining the data from field studies across the season, year and sites with laboratory-based studies using isolates will provide more reliable information regarding the probable environmental impacts of toxins in the natural ecosystem [216]. Species at different ages and different stages of their life cycles should be studied. However, it should be ensured that cultures of similar age and densities are used in comparisons to make a general conclusion at each time point; otherwise, the interpretation of the data may be difficult.

Toxic cyanobacterial species have the potential to dominate the freshwater ecosystems under various environmental conditions [216,217,218], generating the need to improve the current knowledge about co-occurring toxins through exposing aquatic biota to a mixture of toxins, aside from the individual toxin exposure. As some of these studies have already shown, the responses to mixed toxin exposure significantly differ from the exposures in single that more closely represented realistic environmental scenarios. A fundamental understanding of the factors enabling the coexistence of species would be necessary to understand how ecologically niche differentiation influences competition. Niche differences are indeed highly necessary for competing species to establish a stable and long-term coexistence.

From the current literature, pure toxins, toxin containing crude extracts, filtrates of single and mixed cultures as well as the intact cells of the toxin producers induced different physiological and metabolic responses [25,94,219]. Another essential consideration for intraspecies interaction studies is the contribution of other secondary metabolites in addition to cyanotoxins. Metabolic profiles also were different among the species grown in mono- versus co-cultures [220]. Direct co-cultivation approaches, such as membrane-separated technique [91,94], as a more realistic, practical method that mimics natural conditions, are needed to study the molecular basis for cell-cell interactions and advance our knowledge of the subject interspecies interactions in detail but with less interference. Finally, a better understanding of the biotic interactions may help to improve the current approaches towards the mitigation of toxic blooms [35]. 

## Data Availability

Data are available from the corresponding author upon reasonable request.

## References

[1] Van Goethem M.W., Makhalanyane T.P., Cowan D.A., Valverde A. (2017). Cyanobacteria and alphaproteobacteria may facilitate cooperative interactions in niche communities. Front. Microbiol..

[2] Erhard D., Reigosa M.J., Nuria P., González L. (2006). Allelopathy in aquatic environments. Allelopathy: A Physiological Process with Ecological Implications.

[3] Protasov A., Barinova S., Novoselova T., Sylaieva A. (2019). The aquatic organisms diversity, community structure, and environmental conditions. Diversity.

[4] Teittinen A., Virta L., Li M., Wang J. (2021). Factors influencing the biodiversity of three microbial groups within and among islands of the Baltic Sea. FEMS Microbiol. Ecol..

[5] Yang J.R., Yu X., Chen H., Kuo Y.-M., Yang J. (2021). Structural and functional variations of phytoplankton communities in the face of multiple disturbances. J. Environ. Sci..

[6] Zastepa A., Miller T.R., Watson L.C., Kling H., Watson S.B. (2021). Toxins and other bioactive metabolites in deep chlorophyll layers containing the cyanobacteria *Planktothrix* cf. *isothrix* in two Georgian Bay Embayments, Lake Huron. Toxins.

[7] Lawrence D., Fiegna F., Behrends V., Bundy J.G., Phillimore A.B., Bell T., Barraclough T.G. (2012). Species interactions alter evolutionary responses to a novel environment. PLoS Biol..

[8] Odum E.P. (1959). Fundamentals of Ecology.

[9] Lang J.M., Benbow M.E. (2013). Species interactions and competition. Nat. Educ. Knowl..

[10] Kordas R.L., Dudgeon S. (2011). Dynamics of species interaction strength in space, time and with developmental stage. Proc. R. Soc. B.

[11] Zhu C.-M., Zhang J.-Y., Guan R., Hale L., Chen N., Li M., Lu Z.-H., Ge Q.-Y., Yang Y.-F., Zhou J.-Z. (2019). Alternate succession of aggregate-forming cyanobacterial genera correlated with their attached bacteria by co-pathways. Sci. Total Environ..

[12] Omidi A., Esterhuizen-Londt M., Pflugmacher S. (2017). Still challenging: The ecological function of the cyanobacterial toxin microcystin—What we know so far. Toxin Rev..

[13] Sukenik A., Quesada A., Salmaso N. (2015). Global expansion of toxic and non-toxic cyanobacteria: Effect on ecosystem functioning. Biodivers. Conserv..

[14] Scholz S.N., Esterhuizen-Londt M., Pflugmacher S. (2017). Rise of toxic cyanobacterial blooms in temperate freshwater lakes: Causes, correlations and possible countermeasures. Toxicol. Environ. Chem..

[15] Havens K.E., Hudnell H.K. (2008). Cyanobacteria blooms: Effects on aquatic ecosystems. Cyanobacterial Harmful Algal Blooms: State of the Science and Research Needs.

[16] Osman O.A., Beier S., Grabherr M., Bertilsson S. (2017). Interactions of freshwater cyanobacteria with bacterial antagonists. Appl. Environ. Microbiol..

[17] Sellner K., Olson M., Kononen K. (1994). Copepod grazing in a summer cyanobacteria bloom in the Gulf of Finland. Hydrobiologia.

[18] Jiang X., Yang W., Zhao S., Liang H., Zhao Y., Chen L., Li R. (2013). Maternal effects of inducible tolerance against the toxic cyanobacterium *Microcystis aeruginosa* in the grazer *Daphnia carinata*. Environ. Pollut..

[19] Svirčev Z., Lalić D., Savić G.B., Tokodi N., Backović D.D., Chen L., Meriluoto J., Codd G.A. (2019). Global geographical and historical overview of cyanotoxin distribution and cyanobacterial poisonings. Arch. Toxicol..

[20] Sommer U., Gliwicz Z.M., Lampert W., Duncan A. (1986). The PEG-model of seasonal succession of planktonic events in fresh waters. Arch. Hydrobiol..

[21] Wang X.-l., Lu Y.-l., He G.-z., Han J.-y., Wang T.-y. (2007). Exploration of relationships between phytoplankton biomass and related environmental variables using multivariate statistic analysis in a eutrophic shallow lake: A 5-year study. J. Environ. Sci..

[22] Wei J., Wang M., Chen C., Wu H., Lin L., Li M. (2020). Seasonal succession of phytoplankton in two temperate artificial lakes with different water sources. Environ. Sci. Pollut. Res..

[23] Jones M.R., Pinto E., Torres M.A., Dörr F., Mazur-Marzec H., Szubert K., Tartaglione L., Dell’Aversano C., Miles C.O., Beach D.G. (2020). Comprehensive database of secondary metabolites from cyanobacteria. BioRxiv.

[24] Hernández-Zamora M., Santiago-Martínez E., Martínez-Jerónimo F. (2021). Toxigenic *Microcystis aeruginosa* (Cyanobacteria) affects the population growth of two common green microalgae. Evidence of other allelopathic metabolites different to cyanotoxins. J. Phycol..

[25] Pawlik-Skowrońska B., Toporowska M., Mazur-Marzec H. (2019). Effects of secondary metabolites produced by different cyanobacterial populations on the freshwater zooplankters *Brachionus calyciflorus* and *Daphnia pulex*. Environ. Sci. Pollut. Res..

[26] Gross E.M., Wolk C.P., Jüttner F. (1991). Fischerellin, a new allelochemical from the freshwater cyanobacterium *Fischerella muscicola*. J. Phycol..

[27] Mason C., Edwards K., Carlson R., Pignatello J., Gleason F., Wood J. (1982). Isolation of chlorine-containing antibiotic from the freshwater cyanobacterium *Scytonema hofmanni*. Science.

[28] Song H., Lavoie M., Fan X., Tan H., Liu G., Xu P., Fu Z., Paerl H.W., Qian H. (2017). Allelopathic interactions of linoleic acid and nitric oxide increase the competitive ability of *Microcystis aeruginosa*. ISME J..

[29] Qian H., Xu J., Lu T., Zhang Q., Qu Q., Yang Z., Pan X. (2018). Responses of unicellular alga *Chlorella pyrenoidosa* to allelochemical linoleic acid. Sci. Total Environ..

[30] Rzymski P., Poniedziałek B., Kokociński M., Jurczak T., Lipski D., Wiktorowicz K. (2014). Interspecific allelopathy in cyanobacteria: Cylindrospermopsin and *Cylindrospermopsis raciborskii* effect on the growth and metabolism of *Microcystis aeruginosa*. Harmful Algae.

[31] Pflugmacher S. (2002). Possible allelopathic effects of cyanotoxins, with reference to microcystin-LR, in aquatic ecosystems. Environ. Toxicol..

[32] Chia M.A., Jankowiak J.G., Kramer B.J., Goleski J.A., Huang I.-S., Zimba P.V., do Carmo Bittencourt-Oliveira M., Gobler C.J. (2018). Succession and toxicity of *Microcystis* and *Anabaena* (*Dolichospermum*) blooms are controlled by nutrient-dependent allelopathic interactions. Harmful Algae.

[33] Wiegand C., Pflugmacher S. (2005). Ecotoxicological effects of selected cyanobacterial secondary metabolites: A short review. Toxicol. Appl. Pharmacol..

[34] Leão P.N., Vasconcelos M.T., Vasconcelos V.M. (2009). Allelopathy in freshwater cyanobacteria. Crit. Rev. Microbiol..

[35] Kaplan A., Harel M., Kaplan-Levy R.N., Hadas O., Sukenik A., Dittmann E. (2012). The languages spoken in the water body (or the biological role of cyanobacterial toxins). Front. Microbiol..

[36] Keating K.I. (1977). Allelopathic influence on blue-green bloom sequence in a eutrophic lake. Science.

[37] Keating K.I. (1978). Blue-green algal inhibition of diatom growth: Transition from mesotrophic to eutrophic community structure. Science.

[38] Suikkanen S., Fistarol G.O., Granéli E. (2004). Allelopathic effects of the Baltic cyanobacteria *Nodularia spumdigena*, *Aphanizomenon flos-aquae* and *Anabaena lemmermannii* on algal monocultures. J. Exp. Mar. Biol. Ecol..

[39] Suikkanen S., Fistarol G.O., Granéli E. (2005). Effects of cyanobacterial allelochemicals on a natural plankton community. Mar. Ecol. Prog. Ser..

[40] Lirås V., Lindberg M., Nyström P., Annadotter H., Lawton L.A., Graf B. (1998). Can ingested cyanobacteria be harmful to the signal crayfish (*Pacifastacus leniusculus*)?. Freshw. Biol..

[41] Lehtiniemi M., Engström-Öst J., Karjalainen M., Kozlowsky-Suzuki B., Viitasalo M. (2002). Fate of cyanobacterial toxins in the pelagic food web: Transfer to copepods or to faecal pellets?. Mar. Ecol. Prog. Ser..

[42] Andersen R.J., Luu H.A., Chen D.Z., Holmes C.F., Kent M.L., Le Blanc M., Williams D.E. (1993). Chemical and biological evidence links microcystins to salmon ‘netpen liver disease’. Toxicon.

[43] Gilbert J.J. (1990). Differential effects of *Anabaena affinis* on cladocerans and rotifers: Mechanisms and implications. Ecology.

[44] Ferrão-Filho A.d.S., Azevedo S.M., DeMott W.R. (2000). Effects of toxic and non-toxic cyanobacteria on the life history of tropical and temperate cladocerans. Freshwat. Biol..

[45] Ghadouani A., Pinel-Alloul B., Prepas E.E. (2003). Effects of experimentally induced cyanobacterial blooms on crustacean zooplankton communities. Freshwat. Biol..

[46] Miles C., Stirling D. (2020). Toxin Mass List, Version 16. https://www.researchgate.net/publication/337258461_Toxin_mass_list_COM_v160_microcystin_and_nodularin_lists_and_mass_calculators_for_mass_spectrometry_of_microcystins_nodularins_saxitoxins_and_anatoxins.

[47] Janssen E.M.-L. (2019). Cyanobacterial peptides beyond microcystins–A review on co-occurrence, toxicity, and challenges for risk assessment. Water Res..

[48] Jones M.R., Pinto E., Torres M.A., Dörr F., Mazur-Marzec H., Szubert K., Tartaglione L., Dell’Aversano C., Miles C.O., Beach D.G. (2021). CyanoMetDB, a comprehensive public database of secondary metabolites from cyanobacteria. Water Res..

[49] Schatz D., Keren Y., Vardi A., Sukenik A., Carmeli S., Borner T., Dittmann E., Kaplan A. (2007). Towards clarification of the biological role of microcystins, a family of cyanobacterial toxins. Environ. Microbiol..

[50] Zilliges Y., Kehr J.C., Meissner S., Ishida K., Mikkat S., Hagemann M., Kaplan A., Borner T., Dittmann E. (2011). The cyanobacterial hepatotoxin microcystin binds to proteins and increases the fitness of *Microcystis* under oxidative stress conditions. PLoS ONE.

[51] Meissner S., Steinhauser D., Dittmann E. (2015). Metabolomic analysis indicates a pivotal role of the hepatotoxin microcystin in high light adaptation of *Microcystis*. Environ. Microbiol..

[52] Tomescu A., Honegger R., Rothwell G. (2008). Earliest fossil record of bacterial–cyanobacterial mat consortia: The early Silurian Passage Creek biota (440 Ma, Virginia, USA). Geobiology.

[53] Paerl H.W. (1977). Role of heterotrophic bacteria in promoting N 2 fixation by *Anabaena* in aquatic habitats. Microb. Ecol..

[54] Woodhouse J.N., Kinsela A.S., Collins R.N., Bowling L.C., Honeyman G.L., Holliday J.K., Neilan B.A. (2016). Microbial communities reflect temporal changes in cyanobacterial composition in a shallow ephemeral freshwater lake. ISME J..

[55] Weiss G., Kovalerchick D., Lieman-Hurwitz J., Murik O., De Philippis R., Carmeli S., Sukenik A., Kaplan A. (2019). Increased algicidal activity of *Aeromonas veronii* in response to *Microcystis aeruginosa: Interspecies* crosstalk and secondary metabolites synergism. Environ. Microbiol..

[56] Jankowiak J.G., Gobler C.J. (2020). The composition and function of microbiomes within *Microcystis* colonies are significantly different than native bacterial assemblages in two North American Lakes. Front. Microbiol..

[57] Weiss G., Kovalerchick D., Murik O., Sukenik A., Kaplan A., Carmeli S. (2019). Secondary metabolites of *Aeromonas veronii* strain A134 isolated from a *Microcystis aeruginosa* bloom. Metabolites.

[58] Louati I., Pascault N., Debroas D., Bernard C., Humbert J.-F., Leloup J. (2015). Structural diversity of bacterial communities associated with bloom-forming freshwater cyanobacteria differs according to the cyanobacterial genus. PLoS ONE.

[59] Yuan L., Zhu W., Xiao L., Yang L. (2009). Phosphorus cycling between the colonial cyanobacterium *Microcystis aeruginosa* and attached bacteria, *Pseudomonas*. Aquat. Ecol..

[60] Zhao G., Du J., Jia Y., Lv Y., Han G., Tian X. (2012). The importance of bacteria in promoting algal growth in eutrophic lakes with limited available phosphorus. Ecol. Eng..

[61] Liu M., Liu L., Chen H., Yu Z., Yang J.R., Xue Y., Huang B., Yang J. (2019). Community dynamics of free-living and particle-attached bacteria following a reservoir *Microcystis* bloom. Sci. Total Environ..

[62] Liu L., Chen H., Liu M., Yang J.R., Xiao P., Wilkinson D.M., Yang J. (2019). Response of the eukaryotic plankton community to the cyanobacterial biomass cycle over 6 years in two subtropical reservoirs. ISME J..

[63] Chun S.-J., Cui Y., Lee C.S., Cho A.R., Baek K., Choi A., Ko S.-R., Lee H.-G., Hwang S., Oh H.-M. (2019). Characterization of distinct cyanoHABs-related modules in microbial recurrent association network. Front. Microbiol..

[64] Chun S.-J., Cui Y., Lee J.J., Choi I.-C., Oh H.-M., Ahn C.-Y. (2020). Network analysis reveals succession of *Microcystis* genotypes accompanying distinctive microbial modules with recurrent patterns. Water Res..

[65] Wang S., Zhao D., Zeng J., Xu H., Huang R., Jiao C., Guo L. (2019). Variations of bacterial community during the decomposition of *Microcystis* under different temperatures and biomass. BMC Microbiol..

[66] Zhu L., Zancarini A., Louati I., De Cesare S., Duval C., Tambosco K., Bernard C., Debroas D., Song L., Leloup J. (2016). Bacterial communities associated with four cyanobacterial genera display structural and functional differences: Evidence from an experimental approach. Front. Microbiol..

[67] Lezcano M.Á., Velázquez D., Quesada A., El-Shehawy R. (2017). Diversity and temporal shifts of the bacterial community associated with a toxic cyanobacterial bloom: An interplay between microcystin producers and degraders. Water Res..

[68] Li J., Li R., Li J. (2017). Current research scenario for microcystins biodegradation–a review on fundamental knowledge, application prospects and challenges. Sci. Total Environ..

[69] Lezcano M.Á., Morón-López J., Agha R., López-Heras I., Nozal L., Quesada A., El-Shehawy R. (2016). Presence or absence of *mlr* genes and nutrient concentrations co-determine the microcystin biodegradation efficiency of a natural bacterial community. Toxins.

[70] Li J., Shimizu K., Akasako H., Lu Z., Akiyama S., Goto M., Utsumi M., Sugiura N. (2015). Assessment of the factors contributing to the variations in microcystins biodegradability of the biofilms on a practical biological treatment facility. Bioresour. Technol..

[71] Ding Q., Liu K., Song Z., Sun R., Zhang J., Yin L., Pu Y. (2020). Effects of microcystin-LR on metabolic functions and structure succession of sediment bacterial community under anaerobic conditions. Toxins.

[72] Yang C., Xia C., Zhou S., Liu Y. (2010). The permeability effect of microcystin-RR on *Escherichia coli* and *Bacillus subtilis*. Chin. Sci. Bull..

[73] Miguéns D., Valério E. (2015). The impact of some microcystins on the growth of heterotrophic bacteria from Portuguese freshwater reservoir. Limnetica.

[74] Tang X., Gao G., Chao J., Wang X., Zhu G., Qin B. (2010). Dynamics of organic-aggregate-associated bacterial communities and related environmental factors in Lake Taihu, a large eutrophic shallow lake in China. Limnol. Oceanogr..

[75] Wilhelm S.W., Farnsley S.E., LeCleir G.R., Layton A.C., Satchwell M.F., DeBruyn J.M., Boyer G.L., Zhu G., Paerl H.W. (2011). The relationships between nutrients, cyanobacterial toxins and the microbial community in Taihu (Lake Tai), China. Harmful Algae.

[76] Su X., Steinman A.D., Tang X., Xue Q., Zhao Y., Xie L. (2017). Response of bacterial communities to cyanobacterial harmful algal blooms in Lake Taihu, China. Harmful Algae.

[77] Cole J.J. (1982). Interactions between bacteria and algae in aquatic ecosystems. Annu. Rev. Ecol. Syst..

[78] Celussi M., Cataletto B. (2007). Annual dynamics of bacterioplankton assemblages in the Gulf of Trieste (Northern Adriatic Sea). Gene.

[79] Xu Z., Te S.H., He Y., Gin K.Y.-H. (2018). The characteristics and dynamics of cyanobacteria–heterotrophic bacteria between two estuarine reservoirs–tropical versus sub-tropical regions. Front. Microbiol..

[80] Li D., Wu N., Tang S., Su G., Li X., Zhang Y., Wang G., Zhang J., Liu H., Hecker M. (2018). Factors associated with blooms of cyanobacteria in a large shallow lake, China. Environ. Sci. Eur..

[81] Xue Y., Chen H., Yang J.R., Liu M., Huang B., Yang J. (2018). Distinct patterns and processes of abundant and rare eukaryotic plankton communities following a reservoir cyanobacterial bloom. ISME J..

[82] Paerl H.W., Fulton R.S., Moisander P.H., Dyble J., Boynton A. (2001). Harmful freshwater algal blooms, with an emphasis on cyanobacteria. Sci. World J..

[83] Filstrup C.T., Hillebrand H., Heathcote A.J., Harpole W.S., Downing J.A. (2014). Cyanobacteria dominance influences resource use efficiency and community turnover in phytoplankton and zooplankton communities. Ecol. Lett..

[84] McClure R.S., Overall C.C., Hill E.A., Song H.-S., Charania M., Bernstein H.C., McDermott J.E., Beliaev A.S. (2018). Species-specific transcriptomic network inference of interspecies interactions. ISME J..

[85] Singh D.P., Tyagi M.B., Kumar A., Thakur J.K., Kumar A. (2001). Antialgal activity of a hepatotoxin-producing cyanobacterium, *Microcystis aeruginosa*. World J. Microbiol. Biotechnol..

[86] Li Y., Li D. (2012). Competition between toxic Microcystis aeruginosa and nontoxic Microcystis wesenbergii with Anabaena PCC7120. J. Appl. Phycol..

[87] Berry M.A., Davis T.W., Cory R.M., Duhaime M.B., Johengen T.H., Kling G.W., Marino J.A., Den Uyl P.A., Gossiaux D., Dick G.J. (2017). Cyanobacterial harmful algal blooms are a biological disturbance to Western Lake Erie bacterial communities. Environ. Microbiol..

[88] Hu Z.q., Liu Y.d., Li D.h. (2004). Physiological and biochemical analyses of microcystin-RR toxicity to the cyanobacterium *Synechococcus elongatus*. Environ. Toxicol..

[89] Do Carmo Bittencourt-Oliveira M., Camargo-Santos D., do Nascimento Moura A., Francisco I.B., dos Santos Dias C.T., Molica R.J.R., Cordeiro-Araújo M.K. (2013). Effects of toxic and non-toxic crude extracts on different *Microcystis* species (Cyanobacteria). Afr. J. Microbiol. Res..

[90] Toporowska M., Mazur-Marzec H., Pawlik-Skowrońska B. (2020). The effects of cyanobacterial bloom extracts on the biomass, Chl-a, MC and other oligopeptides contents in a natural *Planktothrix agardhii* population. Int. J. Environ. Res. Public Health.

[91] Omidi A., Esterhuizen-Londt M., Pflugmacher S. (2019). *Desmodesmus subspicatus* co-cultured with microcystin producing (PCC 7806) and the non-producing (PCC 7005) strains of *Microcystis aeruginosa*. Ecotoxicology.

[92] Ma Z., Fang T., Thring R.W., Li Y., Yu H., Zhou Q., Zhao M. (2015). Toxic and non-toxic strains of *Microcystis aeruginosa* induce temperature dependent allelopathy toward growth and photosynthesis of *Chlorella vulgaris*. Harmful Algae.

[93] Daniel E., Weiss G., Murik O., Sukenik A., Lieman-Hurwitz J., Kaplan A. (2019). The response of *Microcystis aeruginosa* strain MGK to a single or two consecutive H_2_O_2_ applications. Environ. Microbiol. Rep..

[94] Omidi A., Esterhuizen-Londt M., Pflugmacher S. (2019). Interspecies interactions between *Microcystis aeruginosa* PCC 7806 and *Desmodesmus subspicatus* SAG 86.81 in a co-cultivation system at various growth phases. Environ. Int..

[95] Dong J., Li C., Chang M., Dai D., Liu S., Quan B., Zhang Y., Gao Y. (2019). Effects of toxic cyanobacterium *Microcystis aeruginosa* on the morphology of green alga *Chlorella vulgaris*. Ann. Limnol. Int. J. Limnol..

[96] Vardi A., Schatz D., Beeri K., Motro U., Sukenik A., Levine A., Kaplan A. (2002). Dinoflagellate-cyanobacterium communication may determine the composition of phytoplankton assemblage in a mesotrophic lake. Curr. Biol..

[97] Kearns K.D., Hunter M.D. (2000). Green algal extracellular products regulate antialgal toxin production in a cyanobacterium. Environ. Microbiol..

[98] Chen J., Guo R. (2014). Inhibition effect of green alga on cyanobacteria by the interspecies interactions. Int. J. Environ. Sci. Technol..

[99] Harel M., Weiss G., Lieman-Hurwitz J., Gun J., Lev O., Lebendiker M., Temper V., Block C., Sukenik A., Zohary T. (2013). Interactions between *Scenedesmus* and *Microcystis* may be used to clarify the role of secondary metabolites. Environ. Microbiol. Rep..

[100] Gulati R., Demott W. (1997). The role of food quality for zooplankton: Remarks on the state-of-the-art, perspectives and priorities. Freshw. Biol..

[101] Müller-Navarra D.C., Brett M.T., Liston A.M., Goldman C.R. (2000). A highly unsaturated fatty acid predicts carbon transfer between primary producers and consumers. Nature.

[102] Gliwicz Z.M., Lampert W. (1990). Food thresholds in *Daphnia* species in the absence and presence of blue-green filaments. Ecology.

[103] DeMott W.R., Gulati R.D., Van Donk E. (2001). *Daphnia* food limitation in three hypereutrophic Dutch lakes: Evidence for exclusion of large-bodied species by interfering filaments of cyanobacteria. Limnol. Oceanogr..

[104] Wilson A.E., Sarnelle O., Tillmanns A.R. (2006). Effects of cyanobacterial toxicity and morphology on the population growth of freshwater zooplankton: Meta-analyses of laboratory experiments. Limnol. Oceanogr..

[105] Hulot F.D., Carmignac D., Legendre S., Yepremian C., Bernard C. (2012). Effects of microcystin-producing and microcystin-free strains of Planktothrix agardhii on long-term population dynamics of *Daphnia magna*. Ann. Limnol. Int. J. Limnol..

[106] Kurmayer R., Schober E., Tonk L., Visser P.M., Christiansen G. (2011). Spatial divergence in the proportions of genes encoding toxic peptide synthesis among populations of the cyanobacterium *Planktothrix* in European lakes. FEMS Microbiol. Lett..

[107] Schwarzenberger A., Kurmayer R., Martin-Creuzburg D. (2020). Toward disentangling the multiple nutritional constraints imposed by *Planktothrix*: The significance of harmful secondary metabolites and sterol limitation. Front. Microbiol..

[108] Tang H., Hou X., Xue X., Chen R., Zhu X., Huang Y., Chen Y. (2017). *Microcystis aeruginosa* strengthens the advantage of *Daphnia similoides* in competition with *Moina micrura*. Sci. Rep..

[109] Pflugmacher S., Wiegand C., Oberemm A., Beattie K.A., Krause E., Codd G.A., Steinberg C.E. (1998). Identification of an enzymatically formed glutathione conjugate of the cyanobacterial hepatotoxin microcystin-LR: The first step of detoxication. Biochim. Biophys. Acta Gen. Subj..

[110] Gustafsson S., Rengefors K., Hansson L.-A. (2005). Increased consumer fitness following transfer of toxin tolerance to offspring via maternal effects. Ecology.

[111] Sarnelle O., Wilson A.E. (2005). Local adaptation of *Daphnia pulicaria* to toxic cyanobacteria. Limnol. Oceanogr..

[112] Herrera N.A., Echeverri L.F., Ferrao-Filho A.S. (2015). Effects of phytoplankton extracts containing the toxin microcystin-LR on the survival and reproduction of cladocerans. Toxicon.

[113] Herrera N., Palacio J., Echeverri F., Ferrão-Filho A. (2014). Effects of a cyanobacterial bloom sample containing microcystin-LR on the ecophysiology of *Daphnia similis*. Toxicol. Rep..

[114] Freitas E.C., Pinheiro C., Rocha O., Loureiro S. (2014). Can mixtures of cyanotoxins represent a risk to the zooplankton? The case study of *Daphnia magna* Straus exposed to hepatotoxic and neurotoxic cyanobacterial extracts. Harmful Algae.

[115] Smutná M., Babica P., Jarque S., Hilscherová K., Maršálek B., Haeba M., Bláha L. (2014). Acute, chronic and reproductive toxicity of complex cyanobacterial blooms in *Daphnia magna* and the role of microcystins. Toxicon.

[116] Chislock M.F., Sarnelle O., Jernigan L.M., Wilson A.E. (2013). Do high concentrations of microcystin prevent *Daphnia* control of phytoplankton?. Water Res..

[117] Sadler T., von Elert E. (2014). Physiological interaction of *Daphnia* and *Microcystis* with regard to cyanobacterial secondary metabolites. Aquat. Toxicol..

[118] Paes T.A.S.V., Costa I.A.S.d., Silva A.P.C., Eskinazi-Sant’ anna E. (2016). Can microcystins affect zooplankton structure community in tropical eutrophic reservoirs?. Braz. J. Biol..

[119] Zhao S., Wang Y., Li D. (2014). Effects of toxic and non-toxic *Microcystis aeruginosa* in different mixtures with *Scenedesmus obliquus* on growth of *Brachionus calyciflorus*. J. Freshw. Ecol..

[120] Liang Y., Ouyang K., Chen X., Su Y., Yang J. (2017). Life strategy and grazing intensity responses of *Brachionus calyciflorus* fed on different concentrations of microcystin-producing and microcystin-free *Microcystis aeruginosa*. Sci. Rep..

[121] Nandini S., Sánchez-Zamora C., Sarma S. (2019). Toxicity of cyanobacterial blooms from the reservoir Valle de Bravo (Mexico): A case study on the rotifer *Brachionus calyciflorus*. Sci. Total Environ..

[122] Liang Y., Gao T., Shao L., Min Y., Yang J. (2020). Effects of microcystin-LR and nitrite on the lifespan, reproduction, and heat shock responses of rotifer *Brachionus calyciflorus* at different temperatures. Aquat. Sci..

[123] Koski M., Schmidt K., Engström-Öst J., Viitasalo M., Jónasdóttir S., Repka S., Sivonen K. (2002). Calanoid copepods feed and produce eggs in the presence of toxic cyanobacteria *Nodularia spumigena*. Limnol. Oceanogr..

[124] Wilson A.E., Hay M.E. (2007). A direct test of cyanobacterial chemical defense: Variable effects of microcystin-treated food on two *Daphnia pulicaria* clones. Limnol. Oceanogr..

[125] Ferrão-Filho A.d.S., Da Costa S.M., Ribeiro M.G.L., Azevedo S.M. (2008). Effects of a saxitoxin-producer strain of *Cylindrospermopsis raciborskii* (cyanobacteria) on the swimming movements of cladocerans. Environ. Toxicol..

[126] Ger K.A., Teh S.J., Goldman C.R. (2009). Microcystin-LR toxicity on dominant copepods *Eurytemora affinis* and *Pseudodiaptomus forbesi* of the upper San Francisco Estuary. Sci. Total Environ..

[127] Ger K.A., Hansson L.A., Lürling M. (2014). Understanding cyanobacteria-zooplankton interactions in a more eutrophic world. Freshw. Biol..

[128] Ullah H., Nagelkerken I., Goldenberg S.U., Fordham D.A. (2018). Climate change could drive marine food web collapse through altered trophic flows and cyanobacterial proliferation. PLoS Biol..

[129] Amorim C.A., Ulisses C., Moura A.N. (2017). Biometric and physiological responses of *Egeria densa* Planch. cultivated with toxic and non-toxic strains of *Microcystis*. Aquat. Toxicol..

[130] Senavirathna M.J., Muhetaer G., Zhaozhi L., Fujino T. (2020). Allelopathic influence of low concentration *Microcystis aeruginosa* on *Egeria densa* under different light intensities. Chem. Ecol..

[131] Li Q., Gu P., Zhang H., Luo X., Zhang J., Zheng Z. (2020). Response of submerged macrophytes and leaf biofilms to the decline phase of *Microcystis aeruginosa*: Antioxidant response, ultrastructure, microbial properties, and potential mechanism. Sci. Total Environ..

[132] Pei Y., Liu L., Hilt S., Xu R., Wang B., Li C., Chang X. (2018). Root exudated algicide of *Eichhornia crassipes* enhances allelopathic effects of cyanobacteria *Microcystis aeruginosa* on green algae. Hydrobiologia.

[133] Pflugmacher S. (2004). Promotion of oxidative stress in the aquatic macrophyte *Ceratophyllum demersum* during biotransformation of the cyanobacterial toxin microcystin-LR. Aquat. Toxicol..

[134] Chen G., Zheng Z., Bai M., Li Q. (2020). Chronic effects of microcystin-LR at environmental relevant concentrations on photosynthesis of *Typha angustifolia* Linn. Ecotoxicology.

[135] Wang N., Wang C. (2018). Effects of microcystin-LR on the tissue growth and physiological responses of the aquatic plant *Iris pseudacorus* L.. Aquat. Toxicol..

[136] Christensen V.G., Khan E. (2020). Freshwater neurotoxins and concerns for human, animal, and ecosystem health: A review of anatoxin-a and saxitoxin. Sci. Total Environ..

[137] Bouma-Gregson K., Olm M.R., Probst A.J., Anantharaman K., Power M.E., Banfield J.F. (2018). Microbial diversity and metabolic potential in cyanotoxin producing cyanobacterial mats throughout a river network. bioRxiv.

[138] Thomson-Laing G., Puddick J., Laroche O., Fulton S., Steiner K., Heath M.W., Wood S.A. (2020). Broad and fine scale variability in bacterial diversity and cyanotoxin quotas in benthic cyanobacterial mats. Front. Microbiol..

[139] Cerasino L., Salmaso N. (2020). Co-occurrence of anatoxin-a and microcystins in Lake Garda and other deep subalpine lakes. Adv. Oceanogr. Limnol..

[140] Bouma-Gregson K., Kudela R.M., Power M.E. (2018). Widespread anatoxin-a detection in benthic cyanobacterial mats throughout a river network. PLoS ONE.

[141] Li Q., Gu P., Zhang C., Luo X., Zhang H., Zhang J., Zheng Z. (2020). Combined toxic effects of anatoxin-a and microcystin-LR on submerged macrophytes and biofilms. J. Hazard. Mater..

[142] Papenfort K., Bassler B.L. (2016). Quorum sensing signal–response systems in Gram-negative bacteria. Nat. Rev. Microbiol..

[143] Chia M.A., Cordeiro-Araujo M.K., do Carmo Bittencourt-Oliveira M. (2015). Growth and antioxidant response of *Microcystis aeruginosa* (Cyanobacteria) exposed to anatoxin-a. Harmful Algae.

[144] Chia M.A., Cordeiro-Araújo M.K., Lorenzi A.S., do Carmo Bittencourt-Oliveira M. (2016). Does anatoxin-a influence the physiology of *Microcystis aeruginosa* and *Acutodesmus acuminatus* under different light and nitrogen conditions?. Environ. Sci. Pollut. Res..

[145] Chia M.A., Kramer B.J., Jankowiak J.G., Bittencourt-Oliveira M.d.C., Gobler C.J. (2019). The individual and combined effects of the cyanotoxins, anatoxin-a and microcystin-LR, on the growth, toxin production, and nitrogen fixation of prokaryotic and eukaryotic algae. Toxins.

[146] Kearns K., Hunter M. (2001). Toxin-producing *Anabaena flos-aquae* induces settling of *Chlamydomonas reinhardtii*, a competing motile alga. Microb. Ecol..

[147] De Abreu F.Q., da S. Ferrão-Filho A. (2013). Effects of an Anatoxin-a (s)-producing strain of Anabaena spiroides (Cyanobacteria) on the survivorship and somatic growth of two Daphnia similis clones. J. Environ. Prot..

[148] Bownik A., Pawlik-Skowrońska B. (2019). Early indicators of behavioral and physiological disturbances in *Daphnia magna* (Cladocera) induced by cyanobacterial neurotoxin anatoxin-a. Sci. Total Environ..

[149] Schwarzenberger A., Martin-Creuzburg D. (2021). *Daphnia*’s adaptive molecular responses to the cyanobacterial neurotoxin anatoxin-α are maternally transferred. Toxins.

[150] Ha M.-H., Pflugmacher S. (2013). Time-dependent alterations in growth, photosynthetic pigments and enzymatic defense systems of submerged *Ceratophyllum demersum* during exposure to the cyanobacterial neurotoxin anatoxin-a. Aquat. Toxicol..

[151] Ha M.-H., Contardo-Jara V., Pflugmacher S. (2014). Uptake of the cyanobacterial neurotoxin, anatoxin-a, and alterations in oxidative stress in the submerged aquatic plant *Ceratophyllum demersum*. Ecotoxicol. Environ. Saf..

[152] Pratheepa V., Vasconcelos V. (2017). Binding and pharmacokinetics of the sodium channel blocking toxins (Saxitoxin and the Tetrodotoxins). Mini Rev. Med. Chem..

[153] Walker J.R., Novick P.A., Parsons W.H., McGregor M., Zablocki J., Pande V.S., Du Bois J. (2012). Marked difference in saxitoxin and tetrodoxin affinity for the human nociceptive voltage-gated sodium channel (Naᵥ1. 7). Proc. Natl. Acad. Sci. USA.

[154] Trainer V.L., Baden D.G. (1999). High affinity binding of red tide neurotoxins to marine mammal brain. Aquat. Toxicol..

[155] Pomati F., Rossetti C., Calamari D., Neilan B.A. (2003). Effects of saxitoxin (STX) and veratridine on bacterial Na^+^-K^+^ fluxes: A prokaryote-based STX bioassay. Appl. Environ. Microbiol..

[156] Cusick K.D., Sayler G.S. (2013). An overview on the marine neurotoxin, saxitoxin: Genetics, molecular targets, methods of detection and ecological functions. Mar. Drugs.

[157] Zhang X. (2015). Biodiversity of the symbiotic bacteria associated with toxic marine dinoflagellate *Alexandrium tamarense*. JBM.

[158] Laureano-Rosario A.E., McFarland M., Bradshaw II D.J., Metz J., Brewton R.A., Pitts T., Perricone C., Schreiber S., Stockley N., Wang G. (2021). Dynamics of microcystins and saxitoxin in the Indian River Lagoon, Florida. Harmful Algae.

[159] Raudonis R.A. (2007). Bacteria Associated with Paralytic Shellfish Toxin-Producing Strains of *Anabaena circinalis*. Master’s Thesis.

[160] Do Carmo Bittencourt-Oliveira M., Chia M.A., Camargo-Santos D., Dias C.T. (2016). The effect of saxitoxin and non-saxitoxin extracts of *Cylindrospermopsis raciborskii* (Cyanobacteria) on cyanobacteria and green microalgae. J. Appl. Phycol..

[161] Perreault F., Matias M.S., Melegari S.P., de Carvalho Pinto C.R.S., Creppy E.E., Popovic R., Matias W.G. (2011). Investigation of animal and algal bioassays for reliable saxitoxin ecotoxicity and cytotoxicity risk evaluation. Ecotoxicol. Environ. Saf..

[162] Melegari S.P., Perreault F., Moukha S., Popovic R., Creppy E.E., Matias W.G. (2012). Induction to oxidative stress by saxitoxin investigated through lipid peroxidation in Neuro 2A cells and *Chlamydomonas reinhardtii* alga. Chemosphere.

[163] Haney J.F., Sasner J.J., Ikawa M. (1995). Effects of products released by *Aphanizomenon flos-aquae* and purified saxitoxin on the movements of *Daphnia carinata* feeding appendages. Limnol. Oceanogr..

[164] Ferrão-Filho A.d.S., da Silva D.A.C. (2020). Saxitoxin-producing *Raphidiopsis raciborskii* (cyanobacteria) inhibits swimming and physiological parameters in *Daphnia similis*. Sci. Total Environ..

[165] Ferrão-Filho A.d.S., Dias T.M., Pereira U.J., dos Santos J.A.A., Kozlowsky-Suzuki B. (2019). Nutritional and toxicity constraints of phytoplankton from a Brazilian reservoir to the fitness of cladoceran species. Environ. Sci. Pollut. Res..

[166] Ferrão-Filho A.S., Pereira U.J., Vilar M.C., de Magalhães L., Marinho M.M. (2020). Can small-bodied *Daphnia* control *Raphidiopsis raciborskii* in eutrophic tropical lakes? A mesocosm experiment. Environ. Sci. Pollut. Res..

[167] Rangel L.M., Ger K.A., Silva L.H., Soares M.C.S., Faassen E.J., Lürling M. (2016). Toxicity overrides morphology on *Cylindrospermopsis raciborskii* grazing resistance to the calanoid copepod *Eudiaptomus gracilis*. Microb. Ecol..

[168] Santos G.D., Vilar M.C.P., de Oliveira Azevedo S.M.F. (2020). Acute toxicity of neurotoxin-producing *Raphidiopsis* (*Cylindrospermopsis*) *raciborskii* ITEP-A1 (Cyanobacteria) on the neotropical cladoceran *Macrothrix spinosa*. Ecotoxicol. Environ. Contam..

[169] Ferrão-Filho A.d.S., de Abreu S.D., de Oliveira T.A., de Magalhães V.F., Pflugmacher S., da Silva E.M. (2017). Single and combined effects of microcystin and saxitoxin-producing cyanobacteria on the fitness and antioxidant defenses of cladocerans. Environ. Toxicol. Chem..

[170] Moreira C., Azevedo J., Antunes A., Vasconcelos V. (2013). Cylindrospermopsin: Occurrence, methods of detection and toxicology. J. Appl. Microbiol..

[171] Antunes J.T., Leão P.N., Vasconcelos V.M. (2015). *Cylindrospermopsis raciborskii: Review* of the distribution, phylogeography, and ecophysiology of a global invasive species. Front. Microbiol..

[172] Rzymski P., Poniedziałek B. (2014). In search of environmental role of cylindrospermopsin: A review on global distribution and ecology of its producers. Water Res..

[173] Rasmussen J.P., Cursaro M., Froscio S.M., Saint C.P. (2008). An examination of the antibiotic effects of cylindrospermopsin on common gram-positive and gram-negative bacteria and the protozoan *Naegleria lovaniensis*. Environ. Toxicol..

[174] Wormer L., Cirés S., Carrasco D., Quesada A. (2008). Cylindrospermopsin is not degraded by co-occurring natural bacterial communities during a 40-day study. Harmful Algae.

[175] Dziga D., Kokocinski M., Maksylewicz A., Czaja-Prokop U., Barylski J. (2016). Cylindrospermopsin biodegradation abilities of *Aeromonas* sp. isolated from Rusałka Lake. Toxins.

[176] Martínez-Ruiz E.B., Cooper M., Fastner J., Szewzyk U. (2020). Manganese-Oxidizing bacteria isolated from natural and technical systems remove cylindrospermopsin. Chemosphere.

[177] B-Béres V., Vasas G., Dobronoki D., Gonda S., Nagy S.A., Bácsi I. (2015). Effects of cylindrospermopsin producing cyanobacterium and its crude extracts on a benthic green alga—competition or allelopathy?. Mar. Drugs.

[178] Antunes J.T., Leão P.N., Vasconcelos V.M. (2012). Influence of biotic and abiotic factors on the allelopathic activity of the cyanobacterium *Cylindrospermopsis raciborskii* strain LEGE 99043. Microb. Ecol..

[179] Pinheiro C., Azevedo J., Campos A., Loureiro S., Vasconcelos V. (2013). Absence of negative allelopathic effects of cylindrospermopsin and microcystin-LR on selected marine and freshwater phytoplankton species. Hydrobiologia.

[180] Kovács A.W., Tóth V.R., Pálffy K. (2018). The effects of interspecific interactions between bloom forming cyanobacteria and *Scenedesmus quadricauda* (chlorophyta) on their photophysiology. Acta Biol. Hung..

[181] Mello M.M.E., Soares M.C.S., Roland F., Lürling M. (2012). Growth inhibition and colony formation in the cyanobacterium *Microcystis aeruginosa* induced by the cyanobacterium *Cylindrospermopsis raciborskii*. J. Plankton Res..

[182] Chia M.A., Cordeiro-Araújo M.K., Lorenzi A.S., do Carmo Bittencourt-Oliveira M. (2017). Cylindrospermopsin induced changes in growth, toxin production and antioxidant response of *Acutodesmus acuminatus* and *Microcystis aeruginosa* under differing light and nitrogen conditions. Ecotoxicol. Environ. Saf..

[183] Bar-Yosef Y., Sukenik A., Hadas O., Viner-Mozzini Y., Kaplan A. (2010). Enslavement in the water body by toxic *Aphanizomenon ovalisporum*, inducing alkaline phosphatase in phytoplanktons. Curr. Biol..

[184] Dobronoki D., Viktória B., Vasas G., Gonda S., Nagy S.A., Bácsi I. (2019). Potential role of the cellular matrix of *Aphanizomenon* strains in the effects of cylindrospermopsin—an experimental study. J. Appl. Phycol..

[185] Leonard J.A., Paerl H.W. (2005). Zooplankton community structure, micro-zooplankton grazing impact, and seston energy content in the St. Johns river system, Florida as influenced by the toxic cyanobacterium *Cylindrospermopsis raciborskii*. Hydrobiologia.

[186] Nogueira I.C., Saker M.L., Pflugmacher S., Wiegand C., Vasconcelos V.M. (2004). Toxicity of the cyanobacterium *Cylindrospermopsis raciborskii* to *Daphnia magna*. Environ. Toxicol..

[187] Nogueira I.C., Lobo-da-Cunha A., Vasconcelos V.M. (2006). Effects of *Cylindrospermopsis raciborskii* and *Aphanizomenon ovalisporum* (cyanobacteria) ingestion on *Daphnia magna* midgut and associated diverticula epithelium. Aquat. Toxicol..

[188] Dao T.S., Ortiz-Rodríguez R., Do-Hong L.C., Wiegand C. (2013). Non-microcystin and non-cylindrospermopsin producing cyanobacteria affect the biochemical responses and behavior of *Daphnia magna*. Int. Rev. Hydrobiol..

[189] Flores-Rojas N.C., Esterhuizen-Londt M., Pflugmacher S. (2019). Uptake, growth, and pigment changes in *Lemna minor* L. exposed to environmental concentrations of cylindrospermopsin. Toxins.

[190] Flores-Rojas N.C., Esterhuizen-Londt M., Pflugmacher S. (2015). Antioxidative stress responses in the floating macrophyte *Lemna minor* L. with cylindrospermopsin exposure. Aquat. Toxicol..

[191] Flores-Rojas N.C., Esterhuizen M. (2020). Uptake and effects of cylindrospermopsin: Biochemical, physiological and biometric responses in the submerged macrophyte *Egeria densa* Planch. Water.

[192] Chiswell R.K., Shaw G.R., Eaglesham G., Smith M.J., Norris R.L., Seawright A.A., Moore M.R. (1999). Stability of cylindrospermopsin, the toxin from the cyanobacterium, *Cylindrospermopsis raciborskii*: Effect of pH, temperature, and sunlight on decomposition. Environ. Toxicol..

[193] Santos C., Azevedo J., Campos A., Vasconcelos V., Pereira A.L. (2015). Biochemical and growth performance of the aquatic macrophyte *Azolla filiculoides* to sub-chronic exposure to cylindrospermopsin. Ecotoxicology.

[194] Jámbrik K., Máthé C., Vasas G., Bácsi I., Surányi G., Gonda S., Borbély G., M.-Hamvas M. (2010). Cylindrospermopsin inhibits growth and modulates protease activity in the aquatic plants *Lemna minor* L. and *Wolffia arrhiza* (L.) Horkel. Acta Biol. Hung..

[195] Downing S., Banack S., Metcalf J., Cox P., Downing T. (2011). Nitrogen starvation of cyanobacteria results in the production of β-N-methylamino-L-alanine. Toxicon.

[196] Cox P.A., Banack S.A., Murch S.J., Rasmussen U., Tien G., Bidigare R.R., Metcalf J.S., Morrison L.F., Codd G.A., Bergman B. (2005). Diverse taxa of cyanobacteria produce β-N-methylamino-L-alanine, a neurotoxic amino acid. Proc. Natl. Acad. Sci. USA.

[197] Esterhuizen-Londt M., Downing T. (2008). β-N-methylamino-L-alanine (BMAA) in novel South African cyanobacterial isolates. Ecotoxicol. Environ. Saf..

[198] Réveillon D., Séchet V., Hess P., Amzil Z. (2016). Production of BMAA and DAB by diatoms (Phaeodactylum tricornutum, Chaetoceros sp., Chaetoceros calcitrans and, Thalassiosira pseudonana) and bacteria isolated from a diatom culture. Harmful Algae.

[199] Jiang L., Eriksson J., Lage S., Jonasson S., Shams S., Mehine M., Ilag L.L., Rasmussen U. (2014). Diatoms: A novel source for the neurotoxin BMAA in aquatic environments. PLoS ONE.

[200] Lage S., Costa P.R., Moita T., Eriksson J., Rasmussen U., Rydberg S.J. (2014). BMAA in shellfish from two Portuguese transitional water bodies suggests the marine dinoflagellate *Gymnodinium catenatum* as a potential BMAA source. Aquat. Toxicol..

[201] Visser C. (2011). Evaluation of Model Systems for the Study of Protein Association/Incorporation of Beta-Methylamino-L-Alanine (BMAA). Master’s Thesis.

[202] Van Onselen R., Cook N.A., Phelan R.R., Downing T.G. (2015). Bacteria do not incorporate β-N-methylamino-l-alanine into their proteins. Toxicon.

[203] Berntzon L., Erasmie S., Celepli N., Eriksson J., Rasmussen U., Bergman B. (2013). BMAA inhibits nitrogen fixation in the cyanobacterium *Nostoc* sp. PCC 7120. Mar. Drugs.

[204] Downing S., Downing T.G. (2016). The metabolism of the non-proteinogenic amino acid β-N-methylamino-L-alanine (BMAA) in the cyanobacterium *Synechocystis* PCC6803. Toxicon.

[205] Popova A.A., Rasmussen U., Semashko T.A., Govorun V.M., Koksharova O.A. (2018). Stress effects of cyanotoxin β-methylamino-L-alanine (BMAA) on cyanobacterial heterocyst formation and functionality. Environ. Microbiol. Rep..

[206] Lürling M., Faassen E.J., Van Eenennaam J.S. (2011). Effects of the cyanobacterial neurotoxin β-N-methylamino-L-alanine (BMAA) on the survival, mobility and reproduction of *Daphnia magna*. J. Plankton Res..

[207] Faassen E.J., García-Altares M., e Mello M.M., Lürling M. (2015). Trans generational effects of the neurotoxin BMAA on the aquatic grazer *Daphnia magna*. Aquat. Toxicol..

[208] Esterhuizen-Londt M., Wiegand C., Downing T.G. (2015). β-N-methylamino-L-alanine (BMAA) uptake by the animal model, *Daphnia magna* and subsequent oxidative stress. Toxicon.

[209] Al-Sammak M.A., Hoagland K.D., Cassada D., Snow D.D. (2014). Co-occurrence of the cyanotoxins BMAA, DABA and anatoxin-a in Nebraska reservoirs, fish, and aquatic plants. Toxins.

[210] Esterhuizen-Londt M., Pflugmacher S., Downing T. (2011). β-N-Methylamino-L-alanine (BMAA) uptake by the aquatic macrophyte *Ceratophyllum demersum*. Ecotoxicol. Environ. Saf..

[211] Esterhuizen-Londt M., Pflugmacher S., Downing T. (2011). The effect of β-N-methylamino-L-alanine (BMAA) on oxidative stress response enzymes of the macrophyte *Ceratophyllum demersum*. Toxicon.

[212] Downing S., Esterhuizen-Londt M., Downing T.G. (2015). β-N-methylamino-L-alanine (BMAA) metabolism in the aquatic macrophyte *Ceratophyllum demersum*. Ecotoxicol. Environ. Saf..

[213] Contardo-Jara V., Kuehn S., Pflugmacher S. (2015). Single and combined exposure to MC-LR and BMAA confirm suitability of *Aegagropila linnaei* for use in green liver systems^®^–A case study with cyanobacterial toxins. Aquat. Toxicol..

[214] Lemaire V., Brusciotti S., van Gremberghe I., Vyverman W., Vanoverbeke J., De Meester L. (2012). Genotype× genotype interactions between the toxic cyanobacterium *Microcystis* and its grazer, the waterflea *Daphnia*. Evol. Appl..

[215] Dziallas C., Grossart H.P. (2011). Temperature and biotic factors influence bacterial communities associated with the cyanobacterium *Microcystis* sp.. Environ. Microbiol..

[216] Wiltsie D., Schnetzer A., Green J., Vander Borgh M., Fensin E. (2018). Algal blooms and cyanotoxins in Jordan Lake, North Carolina. Toxins.

[217] Graham J.L., Loftin K.A., Meyer M.T., Ziegler A.C. (2010). Cyanotoxin mixtures and taste-and-odor compounds in cyanobacterial blooms from the Midwestern United States. Environ. Sci. Technol..

[218] Christensen V.G., Maki R.P., Stelzer E.A., Norland J.E., Khan E. (2019). Phytoplankton community and algal toxicity at a recurring bloom in Sullivan Bay, Kabetogama Lake, Minnesota, USA. Sci. Rep..

[219] Esterhuizen-Londt M., Von Schnehen M., Kühn S., Pflugmacher S. (2016). Oxidative stress responses in the animal model, *Daphnia pulex* exposed to a natural bloom extract versus artificial cyanotoxin mixtures. Aquat. Toxicol..

[220] Briand E., Reubrecht S., Mondeguer F., Sibat M., Hess P., Amzil Z., Bormans M. (2019). Chemically mediated interactions between *Microcystis* and *Planktothrix*: Impact on their growth, morphology and metabolic profiles. Environ. Microbiol..

